# Targeted metabolomics analysis of serum and *Mycobacterium tuberculosis* antigen-stimulated blood cultures of pediatric patients with active and latent tuberculosis

**DOI:** 10.1038/s41598-022-08201-4

**Published:** 2022-03-08

**Authors:** Druszczynska Magdalena, Seweryn Michal, Sieczkowska Marta, Kowalewska-Pietrzak Magdalena, Pankowska Anna, Godkowicz Magdalena, Szewczyk Rafał

**Affiliations:** 1 Department of Immunology and Infectious Biology, Institute of Microbiology, Biotechnology and Immunology, Faculty of Biology and Environmental Protection, University of Lodz, Banacha 12/16, 90-237 Lodz, Poland; 2 Biobank Lab, Department of Molecular Biophysics, Faculty of Biology and Environmental Protection, University of Lodz, Banacha 12/16, 90-237 Lodz, Poland; 3 Labexperts sp z o. o. Piekarnicza 5, 80-126 Gdansk, Poland; 4 Regional Specialized Hospital of Tuberculosis, Lung Diseases, and Rehabilitation in Lodz, Okolna 181, 91-520 Lodz, Poland

**Keywords:** Mass spectrometry, Metabolomics

## Abstract

Profound tuberculosis (TB)-induced metabolic changes reflected in the blood metabolomic profile may provide an opportunity to identify specific markers of *Mycobacterium tuberculosis* infection. Using targeted liquid chromatography tandem mass spectrometry, we compared the levels of 30 small metabolites, including amino acids and derivatives, and small organic compounds in serum and *M.tb* antigen-stimulated whole blood cultures of active TB children, latent TB (LTBI) children, nonmycobacterial pneumonia (NMP) children, and healthy controls (HCs) to assess their potential as biomarkers of childhood TB. We found elevated levels of leucine and kynurenine combined with reduced concentrations of citrulline and glutamine in serum and blood cultures of TB and LTBI groups. LTBI status was additionally associated with a decrease in valine levels in blood cultures. The NMP metabolite profile was characterized by an increase in citrulline and glutamine and a decrease in leucine, kynurenine and valine concentrations. The highest discriminatory potential for identifying *M.tb* infection was observed for leucine detected in serum and kynurenine in stimulated blood cultures. The use of targeted metabolomics may reveal metabolic changes in *M.tb*-infected children, and the obtained results are a proof of principle of the usefulness of metabolites in the auxiliary diagnosis of TB in children.

## Introduction

Tuberculosis (TB) is a severe infectious disease caused by the intracellular pathogen *Mycobacterium tuberculosis* (*M.tb*). It is estimated that up to 1/4 of the world's population is latently infected with *M.tb* (LTBI), constituting a huge reservoir of virulent mycobacteria in the environment. TB is considered to be one of the most contagious diseases, as evidenced by the high number of TB cases occurring every year in the world. According to data from the World Health Organization (WHO) report, 10 million new TB cases and 1.3 million deaths were recorded in 2019^1^. There were one million new cases and 230,000 deaths from TB among children. In Poland, the TB incidence rate among children up to the age of 14 reaches 1.8 per 100,000^2^.

Pediatric TB contributes significantly to the worldwide TB burden but remains challenging to diagnose due to insufficient detection methods and the lack of child-specific biomarkers. Contemporary diagnostics of TBare are based on the use of many different methods, but none of them is sensitive enough for clinical use in pediatric patients. The gold standard methods include Ziehl-Neelsenorauramine-rhodaminestained smears and culture of acid-fast bacilli on solid or liquid media. However, at least 10^3^ cells of virulent bacteria per milliliter of sputum are required to be able to see the mycobacteria in the microscopic slides^3^. Mycobacterial culture offers higher sensitivity than smear culture, but it is time-consuming and does not seem to be useful for the diagnosis of disseminated or extrapulmonary TB cases. Genetic and serological tests may accelerate the diagnosis of TB; however, there are still limitations in terms of their sensitivity and specificity^4,5^. Neither of the other TB diagnostic tests, the tuberculin skin test (TST) or the interferon-gamma (IFN-γ) release assay (IGRA), can distinguish active TB from latent *M.tb* infection either in adults or in children. The nonspecificity of pediatric TB clinical symptoms and difficulties in obtaining sputum from children make the diagnosis of TB in young patients even more difficult^6^. Recently, metabolomic analysis (metabolomics), defined as aqualitative and quantitative measurement of metabolic reaction components (metabolites), has developed into a potential tool making progress in the identification of new markers of diseases and infections^7–10^.

As *M.tb* infection causes significant changes in the energy and protein metabolism of the host, many studies with the help of mass spectrometry or nuclear magnetic resonance spectroscopy have been used to identify biomarkers of pulmonary and extrapulmonary TB in blood, urine, and breath^11–16^. However, there are still limited data available on metabolic alterations that occur in children with TB^17–20^. Considering that profound TB-induced metabolic changes reflected in the blood metabolic profile may provide an opportunity to identify specific markers of *M.tb* infection, in the present study, we used targeted tandem liquid chromatography–mass spectrometry (LC–MS/MS) to compare the levels of 30 small metabolites in serum and *M.tb* antigen-stimulated blood cultures of active TB children, LTBI children, infectious nonmycobacterial pneumonia (NMP) children and controls without active infection. Using multivariate analyses, we found metabolites accurately discriminating children with active TB from those with LTBI and healthy controls. The most informative metabolites responsible for the distinction were subsequently identified, and their diagnostic performance in classifying pediatric patients with active TB was evaluated.

## Results

### Study subjects

The study group consisted of 236 Polish children (113 girls and 123 boys) aged 2–15 recruited at the Regional Specialized Hospital of Tuberculosis, Lung Diseases, and Rehabilitation in Lodz, Poland. All children were HIV-negative and vaccinated with the Bacillus Calmette-Guerin (BCG) vaccine in accordance with the calendar of preventive vaccinations in force in Poland. None of the children had evidence of being treated with seroids or other immunosuppressive or anti-tubercular drugs at the time of blood sampling. All children underwent standard physical examination and clinical and radiological evaluation, including chest X-ray, skin tuberculin testing, and IGRA (interferon-gamma release assay) testing. Differential microbiological diagnosis, including diagnostic tests for TB, was performed in children with symptoms of lower respiratory tract infection. For this purpose, gastric aspirates or bronchoaspirates collected from the children were examined using the Ziehl-Neelsen staining method, culture in liquid media (BACTEC MGIT 960 system) and solid media (Löwenstein-Jensen) as well as genetic analysis with the use of the GeneXpert MTB/RIF molecular system. Based on a comprehensive analysis of the results of the clinical and microbiological evaluation, the patients were divided into four groups: (1) children with active pulmonary TB (*M.tb* culture positive), (2) children with latent *M.tb* infection (LTBI) (*M.tb* culture-negative, IGRA positive), (3) children with infectious nonmycobacterial pneumonia without latent *M.tb* infection (NMP) (*M.tb* culture-negative, IGRA negative), and (4) healthy controls (HC) with no signs or symptoms of any lung diseases (IGRA negative). The demographic characteristics of the study groups are presented in Table 1. The median age of the TB children was significantly higher than that of the LTBI or HC groups (p < 0.05). There were no significant differences between the groups of the study regarding the sex. The median values of WBC, RBC, PLT counts and other heamatological parameters did not differ significantly between the studied groups, but the medin CRP concentration was significantly higer in the TB patients than in other groups (p < 0.05) (Table 1).

**Table 1:** Demographic characteristics of study subjects.

Description	Groups			
	TB	LTBI	NMP	HC
	Patients with active TB	Latently M.tb-infected individuals	Patients with infectious nonmycobacterial pneumonia	Healthy controls
n	15	52	20	149
**Age**				
Median years (IQR)	15	8	14	7
	(11–16)	(5–12)	(7–16)	(3–11)
**Sex, n**				
M (%)	5 (33%)	24 (46%)	7 (35%)	87 (58%)
F (%)	10 (67%)	28 (54%)	13 (65%)	62 (42%)
WBC, counts/mm^3^	9770	8150	8176	8562
Neutrophils (%)	60.7	43.8	53.1	40.1
Lymphocytes (%)	26.8	42.6	33.7	47.5
Monocytes (%)	9.2	8.0	9.0	8.1
Eosinophils (%)	2.6	4.7	3.6	3.6
Basophils (%)	0.5	0.5	0.6	0.6
RBC, counts/mm^3^	4,630,000	4,730,000	4,720,000	4,780,000
HGB, g/dl	13.1	13.3	12.9	13.2
HCT, %	38.9	39.4	39.2	39.2
MCHC, g/dl	33.6	33.8	33.1	33.7
PLT, counts/mm^3^	348,300	306,800	337,500	308,800
CRP, mg/l	41.9	0.7	12.9	0.8

*CRP* C-reactive protein, *F* female, *HCT* haematocrit, *hGB* haemoglobin, *M* male*, MCHC* men corpuscular haemoglobin concentration, *M.tb Mycobacterium tuberculosis*, *n* number, *PLT* platelets, *RBC* red blood cells, *SD* standard deviation, *TB* tuberculosis, *WBC* white blood cells.

### Differential abundance of individual metabolites in serum and *Mycobacterium tuberculosis* antigen-stimulated whole blood QFT cultures

Mean, median and standard deviation of individual metabolites measured in serum and *Mycobacterium tuberculosis* antigen-stimulated whole blood QFT cultures, together with the p-value (uncorrected) of the Kruskal–Wallis test for equality of location parameter across study groups are presented in Supplementary Tables S1 and S2. Differences in the abundance of the metabolites in serum and *M.tb* antigen-stimulated whole blood QFT cultures between the four study groups were determined by applying one-tailed ANOVA. The results revealed statistically significant differences for two compounds in serum and five compounds in *M.tb*-stimulated supernatants between the HC group and the active TB, LTBI, and NMP groups (Tables 2, 3). In the post hoc analysis, we found that the serum level of leucine (Leu) was significantly higher in the TB and LTBI groups, while it was markedly lower in the NMP group than in the control HC group. In contrast, we noticed a significant decrease in citrulline (Cit) abundance in the TB and LTBI patients and a significant increase in the level of this metabolite in the NMP group compared to the HC individuals (Table 2). The characteristic metabolomic profile of the TB patients in *M.tb*-stimulated whole blood supernatants was characterized by significantly higher levels of kynurenine (Kyn) in both QFT TB1 and QFT TB2 cultures and leucine (Leu) in OFT TB1 culture alone than in the HC group (Table 3). The levels of Cit and valine (Val) in both QFT TB1 and QFT TB2 cultures and glutamine (Gln) in the QFT TB1 tube were significantly lower in the TB group than in the HC group. LTBI status was associated with a decrease in Cit and Gln abundance and an increase in Leu, Kyn, and Val levels in the *M.tb*-stimulated QFT TB1 and/or TB2 cultures compared to the HC group. The NMP group was characterized by higher Cit and Gln abundances; however, the levels of Leu, Kyn, and Val in QFT TB1 and/or TB2 supernatants were reduced compared to those in HC children (Table 3).

**Table 2:** Relative abundance of individual metabolites in serum.

Metabolite	Relative abundance			p value	Adjusted p value
	HC vs. TB	HC vs, LTBI	HC vs. NMP		
**Leucine (Leu)**	2.157	1.251	− 2.807	0.001	**0.015**
**Citrulline (Cit)**	− 1.020	− 1.014	2.199	0.001	**0.015**
Glycyl-L-valine	0.494	0.053	− 0.669	0.024	0.243
Tyrosine (Tyr)	0.645	− 0.930	2.273	0.052	0.393
Neopterin (Neo)	− 0.598	− 0.226	− 4.026	0.079	0.436
Asparagine (Asn)	− 0.429	0.121	2.021	0.087	0.436
Pyroglutamic acid (Glp)	− 2.038	3.654	− 3.595	0.177	0.735
Phenyloalanine (Phe)	6.552	2.150	− 3.551	0.264	0.735
Alanine (Ala)	− 1.575	4.754	− 6.130	0.271	0.735
Glutathione (GSH)	23.781	12.918	37.071	0.301	0.735
Cysteine (Cys)	− 13.784	− 11.677	0.452	0.324	0.735
Inosine (Ino)	− 1.332	− 0.307	0.939	0.359	0.735
Glycine (Gly)	2.657	2.871	− 1.251	0.401	0.735
Tryptophan (Trp)	− 2.401	− 2.828	− 3.055	0.418	0.735
Isoleucine (Ile)	0.977	0.050	− 0.929	0.429	0.735
Hydroxyproline (Hyp)	− 0.268	− 0.236	− 1.203	0.459	0.735
Cystine	0.019	0.007	− 0.004	0.479	0.735
Proline (Pro)	− 2.383	− 0.903	3.031	0.479	0.735
Threonine (Thr)	− 12.344	− 5.636	− 20.555	0.494	0.735
Methionine (Met)	0.257	0.440	− 0.173	0.501	0.735
Serine (Ser)	1.052	1.111	− 0.755	0.514	0.735
Lysine (Lys)	1.024	− 0.744	− 0.542	0.543	0.741
Para-aminobenzoic acid (PABA)	− 0.629	− 2.477	0.632	0.582	0.747
Kynurenine (Kyn)	0.647	− 0.229	− 0.801	0.609	0.747
Gamma-aminobutyric acid (GABA)	0.180	0.043	− 0.199	0.623	0.747
Glutamine (Gln)	0.370	− 1.228	4.076	0.710	0.819
Valine (Val)	− 1.511	− 0.782	− 2.196	0.782	0.868
Arginine (Arg)	− 0.426	− 0.101	− 0.041	0.866	0.928
Pyridoxal	0.00008	− 0.001	− 0.003	0.985	0.985
Isopyridoxal	0.009	− 0.001	− 0.003	0.985	0.985

*HC* *M.tb*-uninfected healthy controls, *LTBI* latently *M.tb*-infected individuals, *NMP* patients with nonmycobacterial pneumonia, *TB* active tuberculosis patients.

**Table 3:** Relative abundance of individual metabolites in *M.tb*-stimulated whole blood QFT cultures.

Metabolite	Relative abundance			p value	Adjusted p value
	HC vs. TB	HC vs. LTBI	HC vs. NMP		
**OFT TB1 culture**					
**Leucine (Leu)**	2.546	0.926	− 4.842	0.0002	**0.006**
**Citrulline (Cit)**	− 0.538	− 0.760	1.863	0.0005	**0.008**
**Kynurenine (Kyn)**	1.607	2.185	− 0.537	0.001	**0.011**
**Glutamine (Gln)**	− 5.745	− 1.186	8.638	0.004	**0.030**
**Valine (Val)**	− 3.535	0.756	− 6.999	0.008	**0.051**
Hydroxyproline (Hyp)	− 1.734	− 0.844	− 1.807	0.014	0.071
Asparagine (Asn)	1.068	− 0.591	3.616	0.019	0.085
Pyroglutamic acid (Glp)	− 5.098	5.937	− 16.195	0.113	0.388
Glycine (Gly)	1.599	5.251	− 2.551	0.128	0.388
Tyrosine (Tyr)	− 0.656	− 1.261	1.507	0.129	0.388
Tryptophan (Trp)	− 2.049	− 2.432	− 4.730	0.181	0.495
Cysteine (Cys)	12.573	− 4.014	− 16.062	0.207	0.519
Glutathione (GSH)	− 5.396	− 10.880	80.278	0.265	0.613
Threonine (Thr)	8.288	11.717	− 45.299	0.310	0.666
Para-aminobenzoic acid (PABA)	− 5.494	− 5.937	8.398	0.337	0.674
Gamma-aminobutyric acid (GABA)	− 0.140	− 0.152	− 0.346	0.388	0.727
Methionine (Met)	0.616	0.093	− 0.277	0.436	0.770
Lysine (Lys)	− 0.303	0.216	3.012	0.477	0.796
Phenyloalanine (Phe)	4.541	1.442	− 1.869	0.567	0.882
Neopterin (Neo)	− 0.415	0.577	− 1.064	0.600	0.882
Inosine (Ino)	0.032	0.162	0.037	0.617	0.882
Glycyl-L-valine	− 0.052	0.157	− 0.185	0.674	0.919
Alanine (Ala)	− 4.594	− 2.091	− 5.405	0.775	0.999
Proline (Pro)	1.624	0.734	1.981	0.839	0.999
Serine (Ser)	0.715	− 0.001	− 1.120	0.853	0.999
Isoleucine (Ile)	0.502	0.089	− 0.077	0.966	0.999
Cystine	− 0.002	− 0.002	− 0.002	0.972	0.999
Arginine (Arg)	− 0.00001	− 0.00001	0.00008	0.999	0.999
Pyridoxal	− 0.00001	− 0.00001	0.00008	0.999	0.999
Isopyridoxal	− 0.00001	− 0.00001	0.00008	0.999	0.999
**OFT TB2 culture**					
**Kynurenine (Kyn)**	1.153	2.069	− 1.660	0.001	**0.021**
**Citrulline (Cit)**	− 0.562	− 0.800	1.604	0.001	**0.021**
**Valine (Val)**	− 4.365	0.372	− 7.432	0.002	**0.021**
Cysteine (Cys)	27.097	− 1.143	− 16.903	0.013	0.077
Glutamine (Gln)	− 3.741	0.750	10.006	0.013	0.077
Asparagine (Asn)	0.400	− 0.820	2.905	0.015	0.077
Leucine (Leu)	1.088	1.294	− 3.637	0.019	0.085
Hydroxyproline (Hyp)	− 1.784	− 0.771	− 1.519	0.022	0.085
Tyrosine (Tyr)	− 0.546	− 1.178	2.601	0.029	0.098
Pyroglutamic acid (Glp)	− 4.660	2.218	− 14.397	0.038	0.115
Proline (Pro)	3.136	0.538	5.688	0.134	0.366
Glycyl-L-valine	0.172	0.241	− 0.283	0.196	0.492
Isoleucine (Ile)	1.865	0.197	− 0.727	0.241	0.511
Tryptophan (Trp)	− 1.625	− 0.923	− 4.933	0.246	0.511
Glutathione (GSH)	− 19.713	− 16.311	65.044	0.258	0.511
Methionine (Met)	0.818	0.047	0.164	0.272	0.511
Threonine (Thr)	− 11.768	12.204	− 38.171	0.308	0.544
Para-aminobenzoic acid (PABA)	− 5.045	− 5.524	7.459	0.364	0.607
Glycine (Gly)	6.974	2.853	− 0.150	0.394	0.622
Neopterin (Neo)	− 0.453	0.659	− 1.409	0.519	0.779
Lysine (Lys)	2.112	0.218	1.581	0.551	0.787
Phenyloalanine (Phe)	4.061	2.007	− 1.519	0.597	0.815
Inosine (Ino)	− 0.059	0.110	0.027	0.738	0.936
Gamma-aminobutyric acid (GABA)	− 0.052	− 0.106	− 0.288	0.749	0.936
Alanine (Ala)	4.660	1.764	− 3.009	0.797	0.945
Serine (Ser)	0.822	0.022	− 1.066	0.819	0.945
Arginine (Arg)	− 0.005	− 0.005	− 0.005	0.933	0.999
Cystine	− 0.0007	− 0.0008	0.002	0.982	0.999
Pyridoxal	0.0001	0.000001	0.00003	0.999	0.999
Isopyridoxal	0.0001	0.000001	0.00003	0.999	0.999

*HC* M.tb-uninfected healthy controls, *LTBI* latently M.tb-infected individuals, *NMP* patients with nonmycobacterial pneumonia, *TB* active tuberculosis patients.

We visualized the concentration of the metabolites selected for the LASSO analysis by means of the heatmaply package in R (Supplementary Figs. 1–12).

### Discriminative power of metabolites evaluated by receiver operating characteristic (ROC) analysis

For the evaluation of the potential of metabolites measured either in serum *or M.tb*-stimulated whole blood QFT cultures enabling discrimination between the study groups, receiver operating characteristic curves were plotted (Supplementary Figs. 13–24), and the area under the curve (AUC) was determined for each metabolite and the following comparisons: (1) HC vs. TB + LTBI + NMP, (2) TB vs. LTBI + NMP + HC, (3) TB + LTBI vs. HC + NMP, and (4) TB + NMP vs. HC + LTBI (Tables 4, 5). Among the metabolites measured in the serum, glutamine (Gln), threonine (Thr), and pyroglutamic acid (Glp) were characterized by the highest AUC values for TB vs. LTBI + NMP + HC comparison (Table 3). Leucine (Leu) and isoleucine (Ile) showed the highest AUCs for TB + LTBI vs. HC + NMP comparison, whereas the highest AUC values for TB + NMP vs. HC + LTBI differentiation were observed for Leu, hydroxyproline (Hyp) and valine (Val). In HC vs. TB + LTBI + NMP discrimination, the highest AUC value was found for asparagine (Asn) (Table 4).

**Table 4:** Results of the ROC analyses of metabolites measured in sera.

Metabolite	AUC			
	HC vs TB + LTBI + NMP	TB vs. LTBI + NMP + HC	TB + LTBI vs HC + NMP	TB + NMP vs HC + LTBI
Lysine (Lys)	0.556	0.581	0.594	0.594
Arginine (Arg)	0.515	0.544	0.499	0.507
Cystine	0.530	0.482	0.541	0.496
Asparagine (Asn)	0.600	0.602	0.570	0.482
Glycine (Gly)	0.553	0.540	0.571	0.538
Serine (Ser)	0.538	0.496	0.544	0.499
Alanine (Ala)	0.532	0.521	0.550	0.487
Glutamine (Gln)	0.503	0.681	0.557	0.503
Hydroxyproline (Hyp)	0.490	0.607	0.525	0.616
Threonine (Thr)	0.500	0.663	0.546	0.504
Cysteine (Cys)	0.530	0.570	0.511	0.573
Proline (Pro)	0.552	0.506	0.521	0.558
Valine (Val)	0.537	0.556	0.589	0.607
Methionine (Met)	0.559	0.567	0.574	0.547
Tyrosine (Tyr)	0.578	0.525	0.473	0.588
Leucine (Leu)	0.549	0.474	0.620	0.622
Isoleucine (Ile)	0.557	0.525	0.607	0.584
Phenyloalanine (Phe)	0.524	0.514	0.561	0.563
Tryptophan (Trp)	0.521	0.580	0.503	0.574
Glutathione (GSH)	0.532	0.469	0.519	0.544
Gamma-aminobutyric acid (GABA)	0.590	0.472	0.596	0.505
Para-aminobenzoic acid (PABA)	0.519	0.569	0.593	0.584
Kynurenine (Kyn)	0.526	0.545	0.510	0.556
Inosine (Ino)	0.500	0.623	0.474	0.480
Citrulline (Cit)	0.479	0.540	0.420	0.573
Neopterin (Neo)	0.506	0.554	0.527	0.561
Pyridoxal	0.500	0.500	0.500	0.500
Pyroglutamic acid (Glp)	0.497	0.626	0.521	0.605
Isopyridoxal	0.500	0.500	0.500	0.500
Glycyl-L-valine	0.536	0.545	0.552	0.504

*AUC* area under curve, *HC* M.tb-uninfected healthy controls, *LTBI* latently M.tb infected individuals, *NMP* patients with nonmycobacterial pneumonia, *TB* active tuberculosis patients.

**Table 5:** Results of the ROC analyses of metabolites measured in *M.tb*-stimulated whole blood QFT cultures.

Metabolite	AUC			
	HC vs TB + LTBI + NMP	TB vs. LTBI + NMP + HC	TB + LTBI vs. HC + NMP	TB + NMP vs. HC + LTBI
**OFT TB1 culture**				
Lysine (Lys)	0.494	0.535	0.548	0.587
Arginine (Arg)	0.500	0.500	0.500	0.500
Cystine	0.496	0.502	0.496	0.497
Asparagine (Asn)	0.522	0.511	0.510	0.563
Glycine (Gly)	0.556	0.522	0.570	0.522
Serine (Ser)	0.515	0.499	0.531	0.477
Alanine (Ala)	0.504	0.539	0.518	0.542
Glutamine (Gln)	0.536	0.581	0.476	0.565
Hydroxyproline (Hyp)	0.552	0.727	0.522	0.749
Threonine (Thr)	0.509	0.610	0.492	0.562
Cysteine (Cys)	0.511	0.567	0.505	0.546
Proline (Pro)	0.586	0.597	0.474	0.666
Valine (Val)	0.518	0.573	0.487	0.593
Methionine (Met)	0.546	0.655	0.573	0.545
Tyrosine (Tyr)	0.508	0.546	0.524	0.468
Leucine (Leu)	0.532	0.461	0.590	0.567
Isoleucine (Ile)	0.569	0.564	0.578	0.531
Phenyloalanine (Phe)	0.546	0.483	0.569	0.519
Tryptophan (Trp)	0.518	0.549	0.496	0.553
Glutathione (GSH)	0.509	0.507	0.473	0.558
Gamma-aminobutyric acid (GABA)	0.592	0.562	0.538	0.640
Para-aminobenzoic acid (PABA)	0.557	0.547	0.622	0.570
Kynurenine (Kyn)	0.638	0.508	0.717	0.595
Inosine (Ino)	0.587	0.573	0.537	0.563
Citrulline (Cit)	0.491	0.530	0.432	0.578
Neopterin (Neo)	0.511	0.553	0.517	0.534
Pyridoxal	0.500	0.500	0.500	0.500
Pyroglutamic acid (Glp)	0.510	0.579	0.474	0.601
Isopyridoxal	0.500	0.500	0.500	0.500
Glycyl-L-valine	0.503	0.593	0.504	0.546
**OFT TB2 culture**				
Lysine (Lys)	0.492	0.529	0.542	0.570
Arginine (Arg)	0.496	0.502	0.496	0.497
Cystine	0.498	0.507	0.489	0.510
Asparagine (Asn)	0.511	0.564	0.570	0.563
Glycine (Gly)	0.577	0.585	0.581	0.552
Serine (Ser)	0.501	0.502	0.516	0.473
Alanine (Ala)	0.507	0.528	0.519	0.466
Glutamine (Gln)	0.506	0.584	0.555	0.561
Hydroxyproline (Hyp)	0.563	0.728	0.514	0.757
Threonine (Thr)	0.531	0.502	0.559	0.537
Cysteine (Cys)	0.531	0.584	0.534	0.454
Proline (Pro)	0.560	0.608	0.497	0.671
Valine (Val)	0.543	0.578	0.511	0.601
Methionine (Met)	0.548	0.629	0.558	0.559
Tyrosine (Tyr)	0.518	0.532	0.522	0.555
Leucine (Leu)	0.531	0.475	0.585	0.570
Isoleucine (Ile)	0.539	0.554	0.541	0.531
Phenyloalanine (Phe)	0.531	0.546	0.555	0.508
Tryptophan (Trp)	0.529	0.565	0.492	0.576
Glutathione (GSH)	0.497	0.524	0.465	0.540
Gamma-aminobutyric acid (GABA)	0.602	0.637	0.576	0.634
Para-aminobenzoic acid (PABA)	0.564	0.547	0.636	0.581
Kynurenine (Kyn)	0.609	0.525	0.688	0.594
Inosine (Ino)	0.545	0.578	0.505	0.535
Citrulline (Cit)	0.484	0.525	0.425	0.582
Neopterin (Neo)	0.504	0.431	0.512	0.547
Pyridoxal	0.500	0.500	0.500	0.500
Pyroglutamic acid (Glp)	0.509	0.598	0.542	0.603
Isopyridoxal	0.500	0.500	0.500	0.500
Glycyl-L-valine	0.535	0.605	0.480	0.587

*AUC* area under curve, *HC* M.tb-uninfected healthy controls, *LTBI* latently M.tb infected individuals, *NMP* patients with nonmycobacterial pneumonia, *TB* active tuberculosis patients.

The ROC analyses of metabolites measured in *M.tb*-stimulated whole blood QFT TB1 and TB2 cultures showed the highest AUCs for Hyp and methionine (Met) in TB v. LTBI + NMP + HC comparison (Table 4). Kynurenine (Kyn) and para-aminobenzoic acid (PABA) had the highest AUC values in TB + LTBI vs. HC + NMP discrimination, whereas in TB + NMP vs. HC + LTBI comparison, the highest AUCs were observed for Hyp and proline (Pro). In HC vs. TB + LTBI + NMP differentiation, the AUC of Kyn was also significantly higher than that of a random assignment (Table 5).

### Identification of metabolite biomarkers most informative for discriminating between studied groups

Using all metabolites with AUC > 0.55, we performed elastic-net logistic regression analysis to identify the best discriminating set of metabolites between the studied groups. The serum biosignature consisting of hydroxyproline (Hyp), proline (Pro), valine (Val), tyrosine (Tyr), citrulline (Cit) and neopterine (Neo) remained the most informative of the TB + NMP versus HC + LTBI comparison under the fivefold cross-validation procedure (Table 6). In the fivefold cross-validation analysis between the HC versus TB + LTBI + NMP groups, we identified asparagine (Asn) as the most informative metabolite in serum, while between the HC + NMP versus TB + LTBI groups, serum leucine (Leu) had the best discriminating potential (Table 6). Additionally, using the elastic-net logistic regression model, we identified a set of three predictors (Hyp, Pro, Val) in the OFT TB1 culture and eight predictors (Asn, Hyp, Pro, Val, Met, Kyn, Glp) in the OFT TB2 culture informative for the TB + NMP versus HC + LTBI comparison. Only one predictor (Kyn) was informative in the OFT TB1 and QFT TB2 cultures for the HC versus TB + LTBI + NMP and TB + LTBI versus HC + NMP comparisons. The small size of the TB group did not allow the selection of the most informative metabolites for the TB versus LTBI + NMP + HC comparison. The results are summarized in Table 6.

**Table 6:** The results of 3 elastic-net logistic regression models.

Metabolite	HC vs. TB + LTBI + NMP	Metabolite	TB + LTBI vs. HC + NMP	Metabolite	TB + NMP vs HC + LTBI
**Serum**					
Lysine (Lys)	–	Lysine (Lys)	–	Lysine (Lys)	–
Asparagine (Asn)	0.0116	Asparagine (Asn)	–	Hydroxyproline (Hyp)	− 0.0425
Glycine (Gly)	–	Glycine (Gly)	–	Cysteine (Cys)	–
Proline (Pro)	–	Alanine (Ala)	–	Proline (Pro)	0.0001
Methionine (Met)	–	Glutamine (Gln)	–	Valine (Val)	− 0.0075
Tyrosine (Tyr)	–	Valine (Val)	–	Tyrosine (Tyr)	0.0105
Isoleucine (Ile)	–	Methionine (Met)	–	Leucine (Leu)	–
Gamma-aminobutyric acid (GABA)	–	Gamma-aminobutyric acid (GABA)	–	Isoleucine (Ile)	–
		Leucine (Leu)	0.0101	Phenyloalanine (Phe)	–
		Isoleucine (Ile)	–	Tryptophan (Trp)	–
		Para-aminobenzoic acid (PABA)	–	Para-aminobenzoic acid (PABA)	–
		Phenyloalanine (Phe)	–	Kynurenine (Kyn)	–
		Glycyl-L-valine	–	Citrulline (Cit)	0.0121
				Neopterin (Neo)	− 0.0093
				Pyroglutamic acid (Glp)	–
**QFT TB1 culture**					
Glycine (Gly)	–	Glycine (Gly)	–	Lysine (Lys)	–
Hydroxyproline (Hyp)	–	Methionine (Met)	–	Asparagine (Asn)	–
Proline (Pro)	–	Leucine (Leu)	–	Glutamine (Gln)	–
Isoleucine (Ile)	–	Isoleucine (Ile)	–	Hydroxyproline (Hyp)	− 0.1882
Gamma-aminobutyric acid (GABA	–	Para-aminobenzoic acid (PABA)	–	Threonine (Thr)	–
para-aminobenzoic acid (PABA)	–	Phenyloalanine (Phe)	–	Proline (Pro)	–
kynurenine (Kyn)	0.0193	Kynurenine (Kyn)	0.0249	Valine (Val)	0.0155
inosine (Ino)	–			Leucine (Leu)	− 0.0024
				Tryptophan (Trp)	–
				Glutathione (GSH)	–
				Gamma-aminobutyric acid (GABA)	–
				Para-aminobenzoic acid (PABA)	–
				Kynurenine (Kyn)	–
				Inosine (Ino)	–
				Citrulline (Cit)	–
				Pyroglutamic acid (Glp)	–
**QFT TB2 culture**					
Glycine (Gly)	–	Asparagine (Asn)	–	Lysine (Lys)	–
Hydroxyproline (Hyp)	-	Glycine (Gly)	–	Asparagine (Asn)	0.0061
Proline (Pro)	–	Glutamine (Gln)	–	Glycine (Gly)	0.0006
Gamma-aminobutyric acid (GABA)	–	Threonine (Thr)	–	Glutamine (Gln)	–
Para-aminobenzoic acid (PABA)	–	Methionine (Met)	–	Hydroxyproline (Hyp)	− 0.3193
Kynurenine (Kyn)	0.0178	Leucine (Leu)	–	Proline (Pro)	0.0337
		Phenyloalanine (Phe)	–	Valine (Val)	− 0.0279
		Gamma-aminobutyric acid (GABA)	–	Methionine (Met)	0.1224
		Para-aminobenzoic acid (PABA)	–	Tyrosine (Tyr)	–
		Kynurenine (Kyn)	0.0243	Leucine (Leu)	–
				Tryptophan (Trp)	–
				Gamma-aminobutyric acid (GABA)	–
				Para-aminobenzoic acid (PABA)	–
				Kynurenine (Kyn)	− 0.0190
				Citrulline (Cit)	–
				Pyroglutamic acid (Glp)	− 0.0034
				Glycyl-l-valine	–

The coefficients represent relative differences in serum expression of respective metabolites between the studied groups (under the optimal lambda parameter for the penalty function). The most informative set of markers was chosen based on the fivefold cross-validation approach. The dashes correspond to noninformative predictors.

*AUC* area under curve, *HC* M.tb-uninfected healthy controls, *LTBI* latently M.tb infected individuals, *NMP* patients with nonmycobacterial pneumonia, *TB* active tuberculosis patients.

To evaluate the discriminative potential of the selected metabolites, we used a model–based unsupervised clustering approach. In brief, for each experimental condition, we selected the metabolites which have ROC > 0.55 for the comparison between TB from all other groups. In what follows, we applied a high dimenstional normal mixture based clustering approach as implemented in package mclust in R. In this procedure, we first estimated the optimal model (number of clusters as well as the parametrization of the high dimensional normal mixture) by means of Bayes Information Criterion and, subsequently, fitted this model to the data. The plots are presented as Supplementary Figs. 25–33. We evaluated the potential to discriminate the TB group from the remaining three by post-hoc analysis of the profile of samples that are assigned to the estimated components of the mixture.

We also performed dimension reduction by means ogf the t-SNE method with the aid of the Rtsne package in R (Supplementary Fig. 34). We used the exact t-SNE algorithm with perplexity equal to 30. In the plot, the circles represent “serum”, triangles “QFT TB1” and rectangles “QFT TB2”. As far as the color coding is concerned: grey represents HC, green LTBI, blue NMP and red TB.

## Discussion

As an intracellular pathogen, *M.tb* affects numerous protein, lipid, and carohydrate metabolism pathways, leading to marked perturbations of host metabolic homeostasis^12–14,21,22^. Profound TB-induced metabolic changes reflected in the blood metabolomic profile may provide an opportunity to identify specific markers of *M.tb* infection. In recent years, enormous progress has been made in the development and optimization of new high-throughput metabolomics/techniques that have revealed many diverse compounds with diagnostic potential, ranging from modified lipids and peptides to simple amino acids. Although they have been shown to be good candidates in the diagnosis of pulmonary and extrapulmonary TB and serve as helpful tools in monitoring the efficacy of antituberculous treatment in a wide variety of adult cohorts, there are still limited data available on metabolic alterations that occur in children with TB^12,23–27^. Addressing this issue, we used LC–MS/MS to analyze metabolite profiles of serum and *M.tb* antigen-stimulated whole blood cultures obtained from Polish pediatric patients with active TB, children with latent *M.tb* infection, community-acquired nonmycobacterial pneumonia (NMP) and uninfected healthy controls (HCs). We compared the effect of *M.tb* infection on the levels of 30 small metabolites, including amino acids and derivatives, dipeptides, tripeptides, nucleosides, and small organic compounds. The mechanisms underlying the strong effects of mycobacterial infection on host amino acid metabolism are still poorly understood. Our results revealed significant differences between the study groups in the levels of two metabolites measured in serum (leucine—Leu and citrulline—Cit) and five compounds assessed in *M.tb*-stimulated blood cultures (Leu, Cit, kynurenine—Kyn, glutamine—Gln, valine—Val). We found elevated levels of leucine and kynurenine combined with reduced concentrations of citrulline and glutamine in both serum and *M.tb*-stimulated blood cultures of the TB and LTBI groups versus the HC patients. LTBI status was additionally associated with a decrease in Val levels in *M.tb-*stimulated whole blood cultures compared to HC subjects. On the other hand, the metabolite profile of the NMP patients was characterized by an increase in Cit and Gln and a decrease in Leu, Kyn and Val concentrations. Among the metabolites, Leu, one of the three nutritrionally essential branched-chain amino acids (BCAAs), showed the best discriminatory potential for identifying *M.tb* infection in serum, while Kyn, a tryptophan (Trp) metabolite, was the best predictor of suchan infection in whole blood cultures stimulated with mycobacterial antigens.

The two most important metabolites that allowed the identification of *M.tb* infection in the studied pediatric cohort were Leu and Kyn. Leu is known to be able to regulate several cellular processes, such asprotein synthesis, tissue regeneration, and metabolism. It has also been described as an important regulator of the mTOR signaling pathway involved in the development of the immune response^28–30^. The immunomodulatory effect of Leu and its metabolites on the adaptive immune response is the activation of complex 1 of the mammalian target of rapamycin (mTORC1), which controls the metabolic reprogramming of effector T lymphocytes during the activation process^31–33^. Delgoffe et al. showed that mTOR-deficient T cells failed to differentiate into T helper 1 (Th1) or Th17 effector cells despite the lack of a deficit in the production of interleukin (IL)-2^30^. Emerging evidence indicates that Leu, driving the activation of mTORC1, is also critical for multiple processes of M1-like macrophage polarization, including the synthesis of macromolecules^14^. A number of studies indicate that inadequate levels of Leu or other BCAAs (Ile or Val) may lead to serious immune impairment^34^. All these data suggest that significant changes in the Leu levels observed in *M.tb*-infected children may result in multiple metabolic dysregulations. Our observations are in line with recent reports pointing to leucine-rich alpha-2-glycoprotein (LRG) as a new marker of TB^35^. LRG, a secretory type 1 acute phase protein, consists of a single polypeptide chain containing 312 amino acid residues, 66 of which are leucines. They are known to play a role in adhesive interactions between lymphocytes and the endothelium by modulating the surface expression of different receptor types, including the transforming growth factor (TGF) receptor, the granulocyte–macrophage colony-stimulating factor (GM-CSF) receptor, and the macrophage colony stimulating factor (M-CSF) receptor. LRG secretion has been found to be upregulated by some proinflammatory cytokines, including IL-6, IL-1β and tumor necrosis factor (TNF)-α, which are known acute-phase protein inducers^36^. Fujimoto et al. found that serum LRG levels were significantly higher in adult TB patients than in healthy controls and decreased one month after anti-TB therapy, suggesting that leucine-containing LRG might be a promising marker of *M.tb* infection.

The potential of the tryptophan (Trp)/Kyn metabolic pathway in the diagnosis of active TB has been acknowledged in some recent studies^13,23,37^. Two enzymes, tryptophan 2,3-dioxygenase (TDO) and indoleamine 2,3-dioxygenase (IDO-1), responsible for the catalysis of Kyn from Trp were found to be involved in host metabolism linked to *M.tb* infection^38^. Kyn and its metabolites perform a variety of biological functions, including dilating blood vessels during inflammation and regulating the immune response. Fallarino et al. showed that Trp reduction and Kyn accumulation induced selective apoptosis of murine Th1 cells but not Th2 cells, leading to an imbalance in the Th response and development of immunopathology^38^. In our study, pediatric TB patients and LTBI individuals showed increased levels of Kyn in *M.tb-*stimulated whole blood cultures. Moreover, we found significantly higher Kyn/Trp ratios in the TB and LTBI groups than in the HC and NMLP cohorts. An increased Kyn/Trp ratio, an estimate of enhanced activity of IDO-1, showed potential as a biomarker for TB diagnosis in adult patients^23^. We confirmed that the Trp/Kyn ratio measured in the *Mtb*-stimulated cultures may be informative in the comparison between TB + LTBI cases and NMP + HC individuals (AUC = 0.65). However, we are uncertain how high the informativness of this ratio might be compared to other metabolite ratios since we do not have a sample size large enough to perform the association analysis of the ratios of any two metabolites. Furthermore, Dutta et al. showed increased levels of quinolinate, a byproduct of the Kyn pathway, in plasma collected from active TB children and found the metabolite to be the best marker to identify anti-TB treatment efficacy^17^.

The analysis of the intensities of the studied metabolites revealed a significant decrease in Cit levels in the *M.tb*-infected children included in the study. Reduced concentrations of Cit in adult TB patients from South Africa were previously found by Weiner et al.^13^. Cit is one of the intermediates of the urea cycle responsible for the majority of nitrogen excretion through conversion of toxic ammonia to urea in the liver^39^. Moreover, Cit, as an alternative source of intracellular arginine (Arg), has been shown to drive multiple immune functions, including microbicidal nitric oxide (NO) production and robust T cell activity^40,41^. Qualls et al. showed that mice unable to convert L-Cit to L-Arg in their hematopoietic compartment produced less NO and were more susceptible to *M.tb* and *M. bovis* BCG infection^42^. Moreover, L-Cit-based T cell metabolism was shown to be essential for the accumulation of CD4 + T cells at the site of infection, confirming that this metabolic pathway was involved in antimycobacterial T cell immunity in vivo^40^. Thus, it may be suggested that decreased Cit levels observed in TB patients may impair the host immune defense against mycobacterial infection.

During infection, host immune cells undergo modifications of metabolic pathways to generate sufficient energy to perform a variety of defense functions. In a process called glutaminolysis, Gln, the most abundant amino acid circulating in the blood, is converted into tricarboxylic acid (TCA) cycle metabolites such as glutamate through the activity of multiple enzymes^43^. In our study, the *M.tb*-infected children showed low Gln levels in comparison with the healthy controls, reflecting both *M.tb* adaptive mechanisms and the host immune response. Lee et al. found that the pathogen converts Gln to glutamate using glutamate synthase to neutralize intracellular pH^44^ and subsequently exploits glutamate for the synthesis of cell wall components during the growth phase^45^. Our results concerning major metabolic changes, which we discovered in serum and mycobacterial antigen-stimulated cultures of children infected with *M.tb,* are in line with the majority of the findings from previous MS studies^13,19,21,23^. Sun et al., Cho et al. and Vrieling et al. reported a significant decrease in Gln levels in active TB patients, and Weiner et al. found low levels of Cit and Gln and high levels of Kyn in patients with active TB in comparison with LTBI subjects and healthy controls. A recent study by Weiner et al. demonstrated that a decrease in Gln constituted one of the prognostic signatures that identified individuals who progress to clinical TB among patients with preclinical TB^24^. Concordantly, Cho et al. demonstrated that the Gln/glutamic acid (Glu) ratio showed good clinical performance in diagnosing active TB^21^.

Despite the interesting results of our research, there were some limitations. The first weakness was the low number of children with active TB; however, we were able to detect highly significant differences in the levels of metabolites in serum or *M.tb* antigen-stimulated blood cultures between the studied cohorts. The active TB group size was too small to reveal the most informative metabolites that would allow differentiating active TB and latent *M.tb* infection. Importantly, we found combinations of metabolites that had the potential to discriminate *M.tb*-associated pneumonia from nonmycobacterial lung disease-associated pneumonia. While these differences may not be sufficient for clinical purposes, the results may prove that some of the studied metabolites may be useful in diagnostic tests. TB also has a well-known association with HIV coinfection, which was not assessed in this study. To validate the findings and prove the potential of the proposed metabolites as diagnostic biomarkers for pediatric *M.* TB, more children with pulmonary TB, as well as children with extrapulmonary TB and immunocompromised children, need to be included in future research.

In conclusion, *M.tb* adaptive strategies and mechanisms of the host immune response can be successfully exploited for the development of new TB diagnostic tools. The use of targeted metabolomics provides insight into metabolic dysregulation in *M.tb*-infected children and paves the way for taking advantage of metabolites potentially useful in the diagnosis of pediatric TB. Our results provide convincing evidence of the need for further research aimed at developing a metabolomic signature of pediatric TB.

## Materials and methods

### Ethics statement

The study was approved by the Research Ethics Committee of Medical University in Lodz (no. RNN/138/15/KE). Written informed consent was obtained from parents or guardians of children included in the study. All methods were performed in accordance with the relevant guidelines and regulations (e.g. Declaration of Helsinki).

### Sample preparation

Serum was derived from clotted blood tubes. For serum collection, SST BD Vacutainer Tubes were used, centrifuged for 10 min at 2500×*g* within 2 h of blood draw, aliquoted and stored at − 70 °C until processed. The QuantiFERON TB Gold Plus (QFT) test (Qiagen, Germany) was performed according to the manufacturer’s instructions. Briefly, blood was drawn by venepuncture and then incubated at 37 °C for 16–24 h with either *M.tb*-specific antigens, i.e., TB1 and TB2, a mitogen or without stimulation (Nil). The cell-free culture supernatants were collected and frozen at − 20 °C until analysis.

Sample preparation was carried out as follows: 1 ml of a mixture of ethanol, acetonitrile and water at a ratio of 60:30:10 was added to 200 µl of serum or QFT supernatant. The mixtures were vortexed for 1 min, incubated for 15 min at 4 °C, and then centrifuged at 4 °C at 10,000 × *g* for 10 min. After protein precipitation, the supernatants were diluted 100 × in a 90% acetonitrile solution with 20 mM ammonium formate and 0.1% formic acid and analyzed immediately using LC–MS/MS.

### LC–MS/MS analysis

LC–MS/MS analysis was conducted on a QTRAP 6500 (Sciex, USA) mass spectrometer coupled with an ExionAC (Sciex, USA) liquid chromatograph. The targeted scheduled multiple reaction monitoring (sMRM) LC–MS/MS method included 23 amino acids and 7 other compounds that may act as potential biomarkers of TB^11–16^. Depending on the nature of the compounds, quantifier and qualifier ions (2 MRM pairs per compound) in positive or negative ionization mode were selected and optimized. Ion source parameters are described in Supplementary Table S3, and compound-dependent parameters are presented in Supplementary Table S4. Chromatographic separation was conducted under HILIC conditions on an Agilent Poroshell 120 HILIC-Z 3.0 × 100 mm 2.7 µm. The major liquid chromatography parameters were as follows: injection volume, 10 µl; flow, 500 µl/min; column temperature: 40 °C. The mobile phase consisted of water (A) and 90% acetonitrile in water (B), both with the addition of 20 mM ammonium formate with 0.1% formic acid. The following gradient elution was applied: 100% B for 1 min, followed by a linear increase to 36% B in the 5th min, reverse initial condition in 5.1 min and column stabilization until the 7th min. The quantitation of the monitored compounds was performed on the basis of standard curves. The following criteria were applied to define working ranges for each monitored compound: limit of detection (LOD)—S/N ≥ 3, lower limit of quantitation (LLOQ)—S/N ≥ 6, linearity—R ≥ 0.995, weighting 1/x. The working range was defined as a linear regression between LLOQ and the highest point of the curve with RR ≥ 0.995 (Supplementary Table S5).

### Statistical analysis

Differential abundance analysis of metabolites was performed using the ‘limma’ package in R. In short, the data were normalized using the quantile normalization procedure. Afterwards, linear models were fitted, where the dependent variable was defined as the metablites’ abundance and the independent variable was the group assignment. After model fitting, appropriate contrasts were selected, and tests were performed to estimate the effect size and assess the p value. FDR was estimated via the empirical Bayes method implementing the package ‘limma’. The ROC curves and AUC values were estimated via the pROC package in R. The multiparametric penalized regression models (elastic-net) were fitted with the aid of the ‘glmnet’ package in R. The lambda (penalty coefficient) was estimated via the fivefold cross-validation method as implemented in the ‘cv.glmnet’ function in R.

## Supplementary Information

Supplementary Information 1.

Supplementary Information 2.
