# Supplementary material for: Targeted metabolomics analysis of serum and Mycobacterium tuberculosis antigen-stimulated blood cultures of pediatric patients with active and latent tuberculosis
Source: Sci Rep. 2022 Mar 8;12:4131. doi: 10.1038/s41598-022-08201-4 (PMC8904507; doi:10.1038/s41598-022-08201-4)
Supplement: Supplementary file 1 — Supplementary Information 1. [file 41598_2022_8201_MOESM1_ESM.pdf]

Supplementary Figures

Targeted metabolomics analysis of serum and *Mycobacterium tuberculosis* antigens-stimulated blood cultures of pediatric patients with active and latent tuberculosis

Druszczynska Magdalena[1], Seweryn Michal[2], Sieczkowska Marta[3], Kowalewska-Pietrzak Magdalena[4], Pankowska Anna[4], Godkowicz Magdalena[1], Szewczyk Rafał[3]

Supplementary Figure 1. Heatmap visualization of metabolites measured in serum. Rows: samples; columns: metabolites. Light blue: TB; dark blue: HC+LTBI+NMP. Colour key indicates metabolite expression value: dark blue: lowest; dark red: highest.

Supplementary Figure 2. Heatmap visualization of metabolites measured in serum. Rows: samples; columns: metabolites. Light blue: TB+LTBI; dark blue: HC+NMP. Colour key indicates metabolite expression value: dark blue: lowest; dark red: highest.

Supplementary Figure 3. Heatmap visualization of metabolites measured in serum. Rows: samples; columns: metabolites. Light blue: TB+NMP; dark blue: HC+LTBI. Colour key indicates metabolite expression value: dark blue: lowest; dark red: highest.

Supplementary Figure 4. Heatmap visualization of metabolites measured in serum. Rows: samples; columns: metabolites. Light blue: HC; dark blue: TB+LTBI+NMP. Colour key indicates metabolite expression value: dark blue: lowest; dark red: highest.

Supplementary Figure 5. Heatmap visualization of metabolites measured in QFT TB1 culture. Rows: samples; columns: metabolites. Light blue: TB; dark blue: HC+LTBI+NMP. Colour key indicates metabolite expression value: dark blue: lowest; dark red: highest.

Supplementary Figure 6. Heatmap visualization of metabolites measured in QFT TB1 culture. Rows: samples; columns: metabolites. Light blue: TB+LTBI; dark blue: HC+NMP. Colour key indicates metabolite expression value: dark blue: lowest; dark red: highest.

Supplementary Figure 7. Heatmap visualization of metabolites measured in QFT TB1 culture. Rows: samples; columns: metabolites. Light blue: TB+NMP; dark blue: HC+LTBI. Colour key indicates metabolite expression value: dark blue: lowest; dark red: highest.

Supplementary Figure 8. Heatmap visualization of metabolites measured in QFT TB1 culture. Rows: samples; columns: metabolites. Light blue: HC; dark blue: TB+LTBI+NMP. Colour key indicates metabolite expression value: dark blue: lowest; dark red: highest.

Supplementary Figure 9. Heatmap visualization of metabolites measured in QFT TB2 culture. Rows: samples; columns: metabolites. Light blue: TB; dark blue: HC+LTBI+NMP. Colour key indicates metabolite expression value: dark blue: lowest; dark red: highest.

Supplementary Figure 10. Heatmap visualization of metabolites measured in QFT TB2 culture. Rows: samples; columns: metabolites. Light blue: TB+LTBI; dark blue: HC+NMP. Colour key indicates metabolite expression value: dark blue: lowest; dark red: highest.

Supplementary Figure 11. Heatmap visualization of metabolites measured in QFT TB2 culture. Rows: samples; columns: metabolites. Light blue: TB+NMP; dark blue: HC+LTBI. Colour key indicates metabolite expression value: dark blue: lowest; dark red: highest.

Supplementary Figure 12. Heatmap visualization of metabolites measured in QFT TB2 culture. Rows: samples; columns: metabolites. Light blue: HC; dark blue: TB+LTBI+NMP. Colour key indicates metabolite expression value: dark blue: lowest; dark red: highest.

Supplementary Figure 13. ROC curves for the metabolites measured in serum for the differentiation of TB patients from other groups.

Supplementary Figure 14. ROC curves for the metabolites measured in serum for the differentiation of *M.tb*-infected individuals (TB+LTBI) from other groups (HC+NMP).

Supplementary Figure 15. ROC curves for the metabolites measured in serum for the differentiation of patients with pneumonia (TB+NMP) from other groups (HC+LTBI).

Supplementary Figure 16. ROC curves for the metabolites measured in serum for the differentiation of healthy controls (HC) from other groups (TB+LTBI+NMP).

Supplementary Figure 17. ROC curves for the metabolites measured in OFT TB1 cultures for the differentiation of TB patients from other groups.

Supplementary Figure 18. ROC curves for the metabolites measured in OFT TB1 cultures for the differentiation of *M.tb*-infected individuals (TB+LTBI) from other groups (HC+NMP).

Supplementary Figure 19. ROC curves for the metabolites measured in OFT TB1 cultures for the differentiation of patients with pneumonia (TB+NMP) from other groups (HC+LTBI)

Supplementary Figure 20. ROC curves for the metabolites measured in OFT TB1 cultures for the differentiation of healthy controls (HC) from other groups (TB+LTBI+NMP).

Supplementary Figure 21. ROC curves for the metabolites measured in OFT TB2 cultures for the differentiation of TB patients from other groups.

Supplementary Figure 22. ROC curves for the metabolites measured in OFT TB2 cultures for the differentiation of *M.tb*-infected individuals (TB+LTBI) from other groups (HC+NMP).

Supplementary Figure 23. ROC curves for the metabolites measured in OFT TB2 cultures for the differentiation of patients with pneumonia (TB+NMP) from other groups (HC+LTBI)

Supplementary Figure 24. ROC curves for the metabolites measured in OFT TB2 cultures for the differentiation of healthy controls (HC) from other groups (TB+LTBI+NMP).

Supplementary Figure 25. Unsupervised model-based clustering results of metabolites measured in serum of TB patients vs HC subjects

Supplementary Figure 26. Unsupervised model-based clustering results of metabolites measured in serum of TB patients vs LTBI subjects

Supplementary Figure 27. Unsupervised model-based clustering results of metabolites measured in serum of TB patients vs NMP patients

Supplementary Figure 28. Unsupervised model-based clustering results of metabolites measured in OFT TB1 cultures of TB patients vs HC subjects

Supplementary Figure 29. Unsupervised model-based clustering results of metabolites measured in OFT TB1 cultures of TB patients vs LTBI subjects

Supplementary Figure 30. Unsupervised model-based clustering results of metabolites measured in OFT TB1 cultures of TB patients vs NMP patients

Supplementary Figure 31. Unsupervised model-based clustering results of metabolites measured in OFT TB2 cultures of TB patients vs HC subjects

Supplementary Figure 32. Unsupervised model-based clustering results of metabolites measured in OFT TB1 cultures of TB patients vs LTBI subjects

Supplementary Figure 33. Unsupervised model-based clustering results of metabolites measured in OFT TB1 cultures of TB patients vs NMP patients

Supplementary Figure 34. Dimension reduction plot by means ogf the t-SNE method with the aid of the Rtsne package in R. We used the exact t-SNE algorithm with perplexity equal to 30. In the plot, the circles represent “serum”, triangles “QFT TB1” and rectangles “QFT TB2”. As far as the color coding is concerned: grey represents HC, green LTBI, blue NMP and red TB.

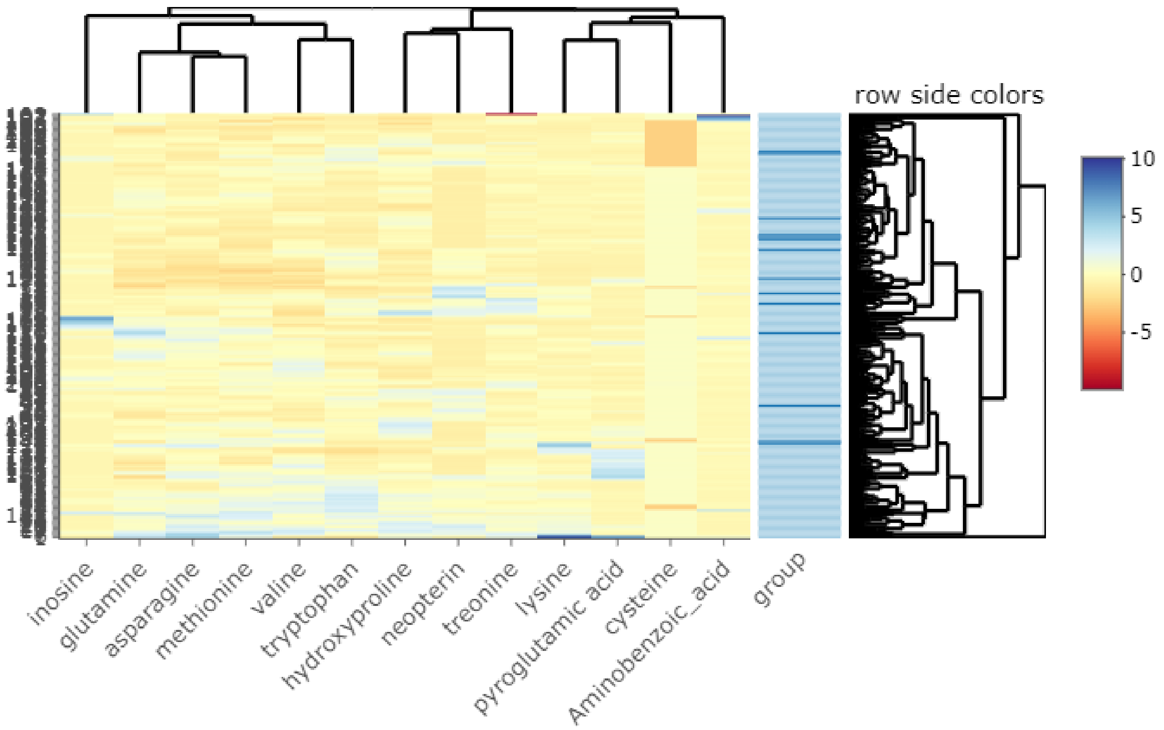

Supplementary Figure 1. Heatmap visualization of metabolites measured in serum. Rows: samples; columns: metabolites. Light blue: TB; dark blue: HC+LTBI+NMP. Colour key indicates metabolite expression value: dark blue: lowest; dark red: highest.

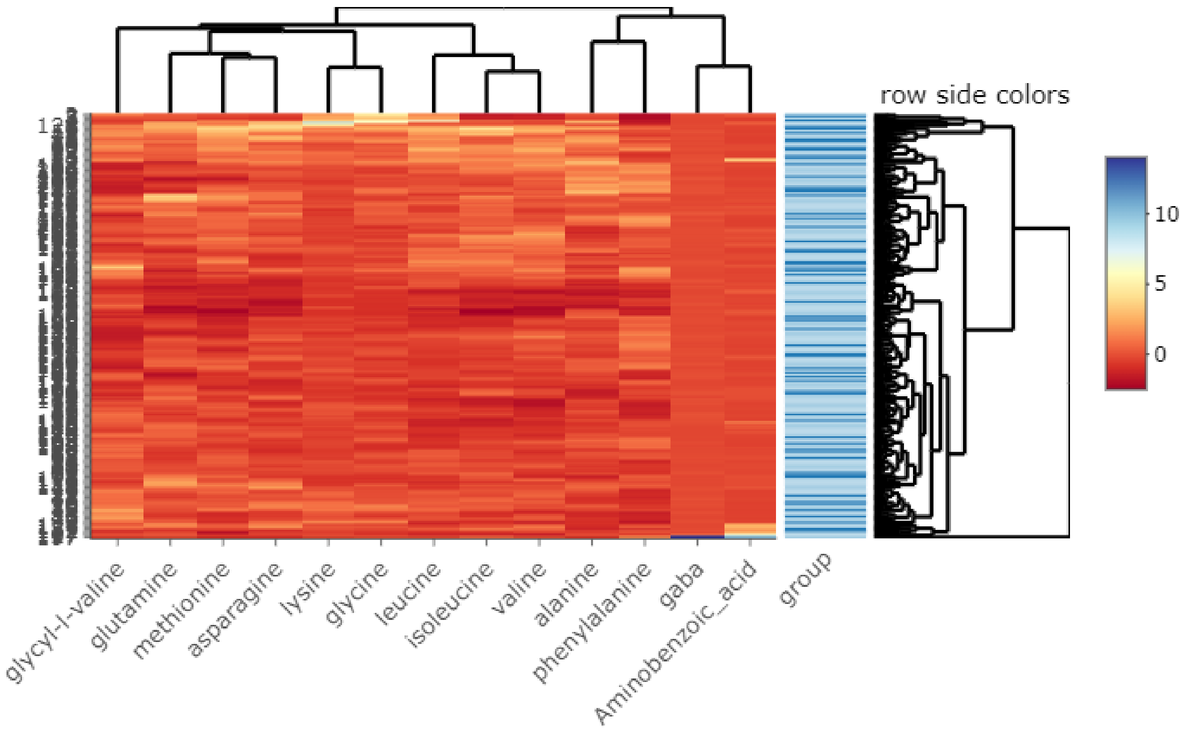

Supplementary Figure 2. Heatmap visualization of metabolites measured in serum. Rows: samples; columns: metabolites. Light blue: TB+LTBI; dark blue: HC+NMP. Colour key indicates metabolite expression value: dark blue: lowest; dark red: highest.

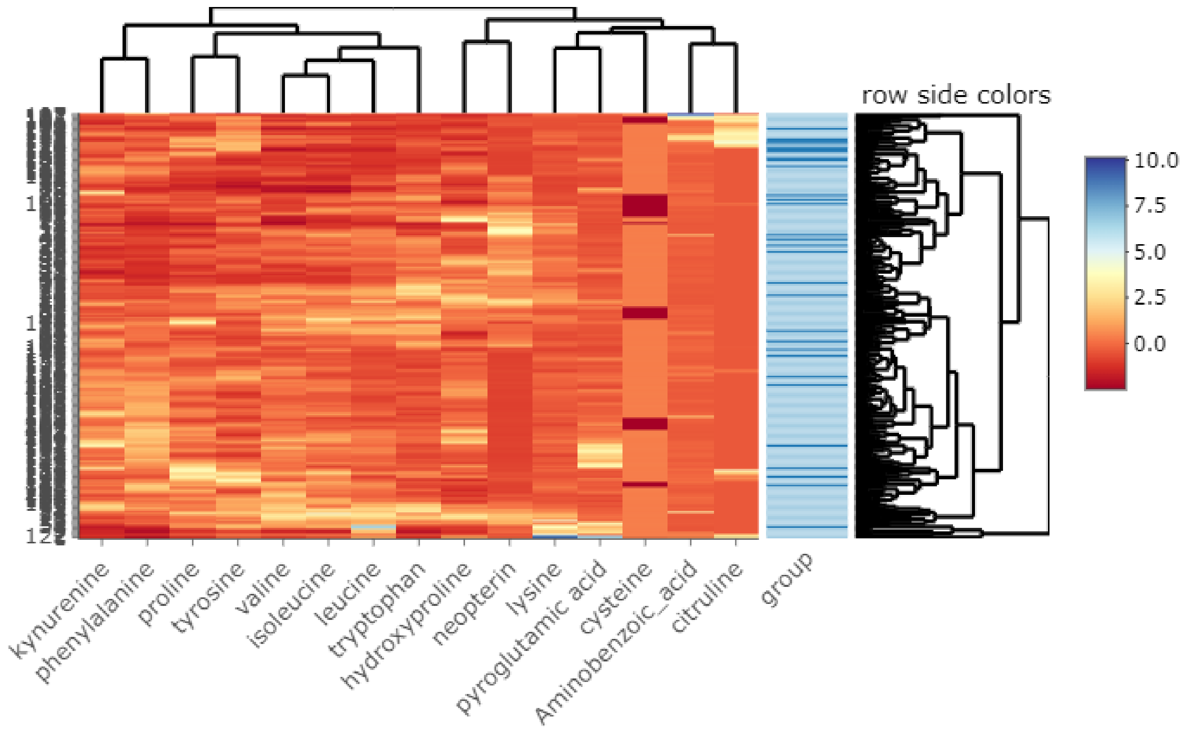

Supplementary Figure 3. Heatmap visualization of metabolites measured in serum. Rows: samples; columns: metabolites. Light blue: TB+NMP; dark blue: HC+LTBI. Colour key indicates metabolite expression value: dark blue: lowest; dark red: highest.

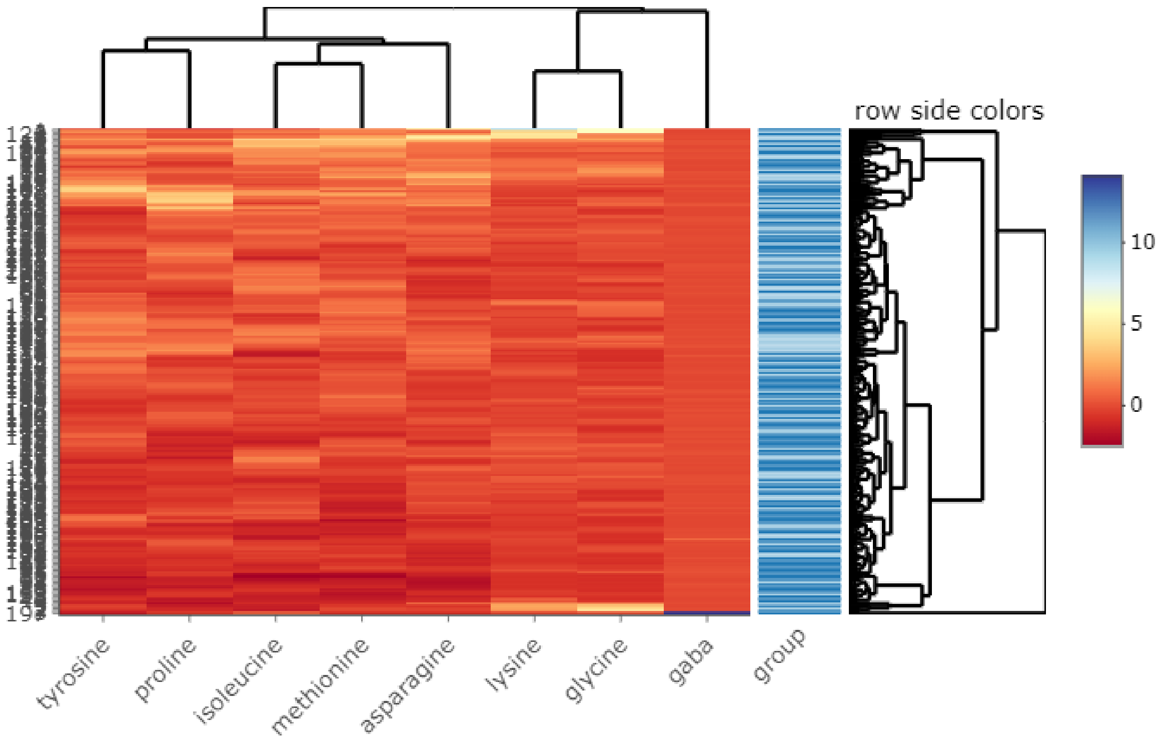

Supplementary Figure 4. Heatmap visualization of metabolites measured in serum. Rows: samples; columns: metabolites. Light blue: HC; dark blue: TB+LTBI+NMP. Colour key indicates metabolite expression value: dark blue: lowest; dark red: highest.

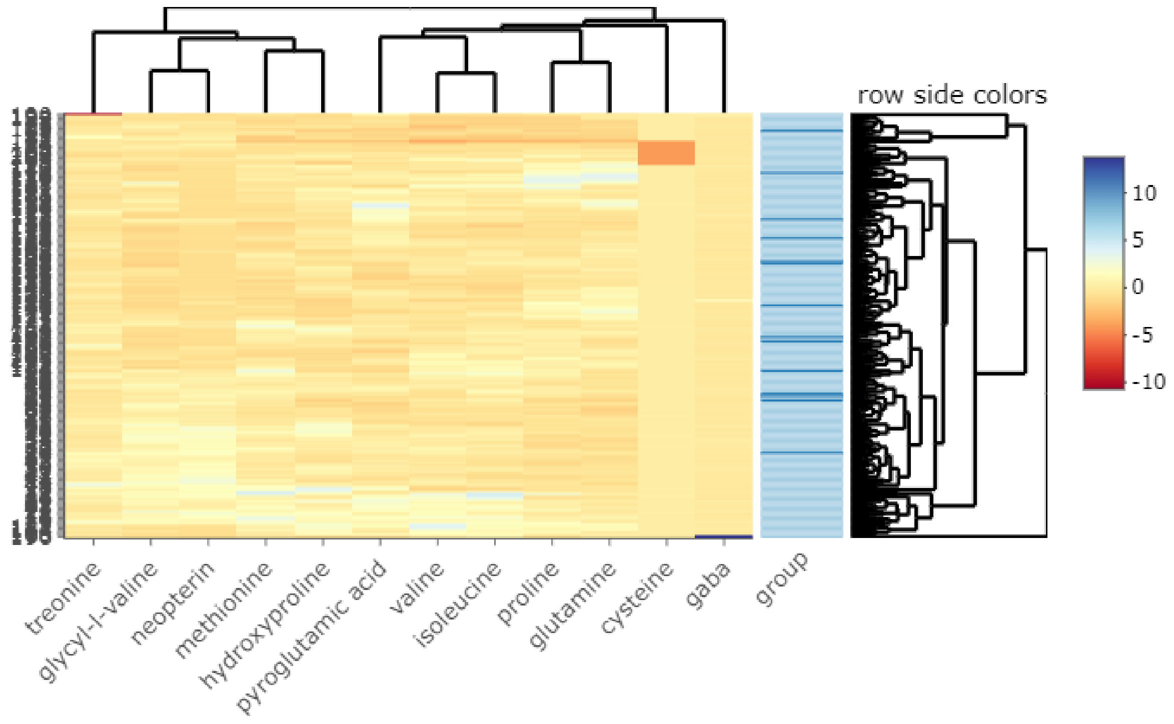

Supplementary Figure 5. Heatmap visualization of metabolites measured in QFT TB1 culture. Rows: samples; columns: metabolites. Light blue: TB; dark blue: HC+LTBI+NMP. Colour key indicates metabolite expression value: dark blue: lowest; dark red: highest.

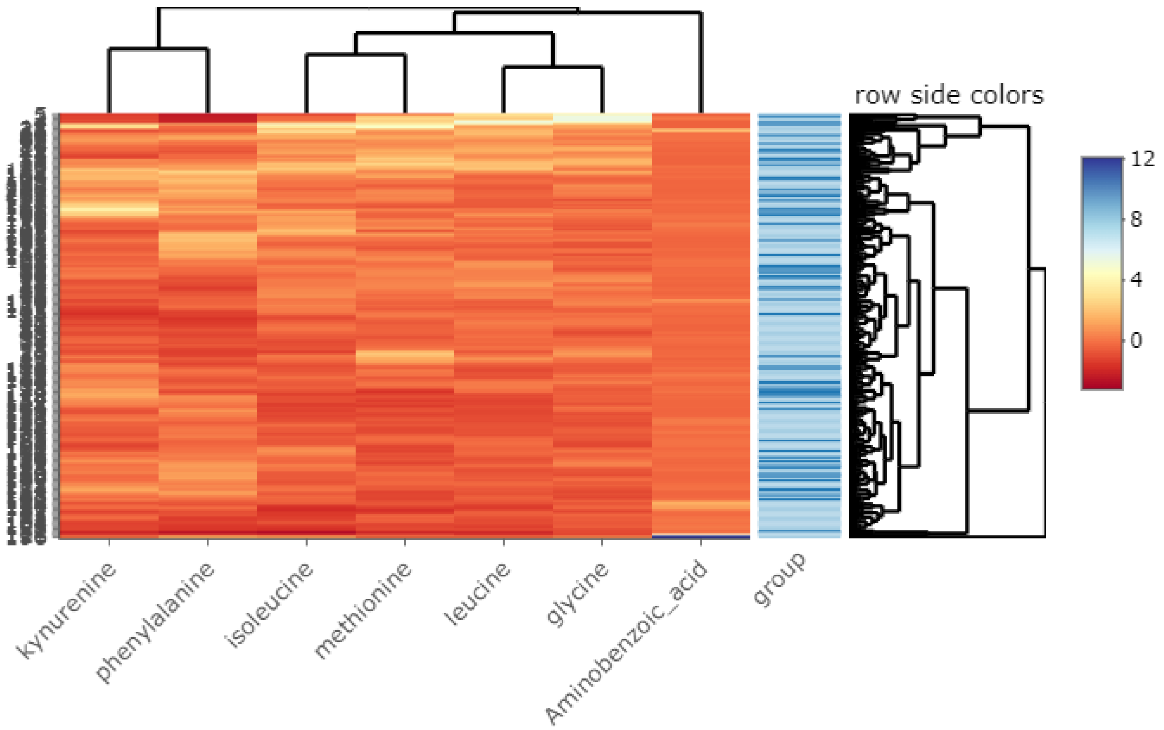

Supplementary Figure 6. Heatmap visualization of metabolites measured in QFT TB1 culture. Rows: samples; columns: metabolites. Light blue: TB+LTBI; dark blue: HC+NMP. Colour key indicates metabolite expression value: dark blue: lowest; dark red: highest.

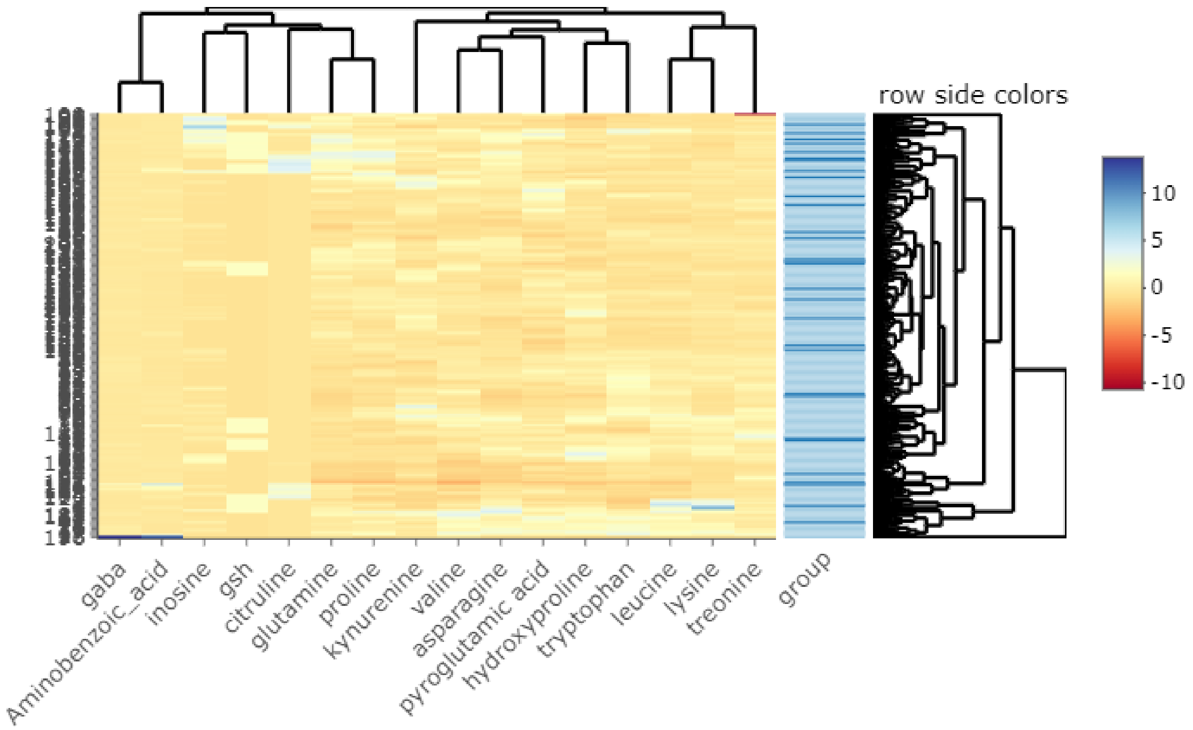

Supplementary Figure 7. Heatmap visualization of metabolites measured in QFT TB1 culture. Rows: samples; columns: metabolites. Light blue: TB+NMP; dark blue: HC+LTBI. Colour key indicates metabolite expression value: dark blue: lowest; dark red: highest.

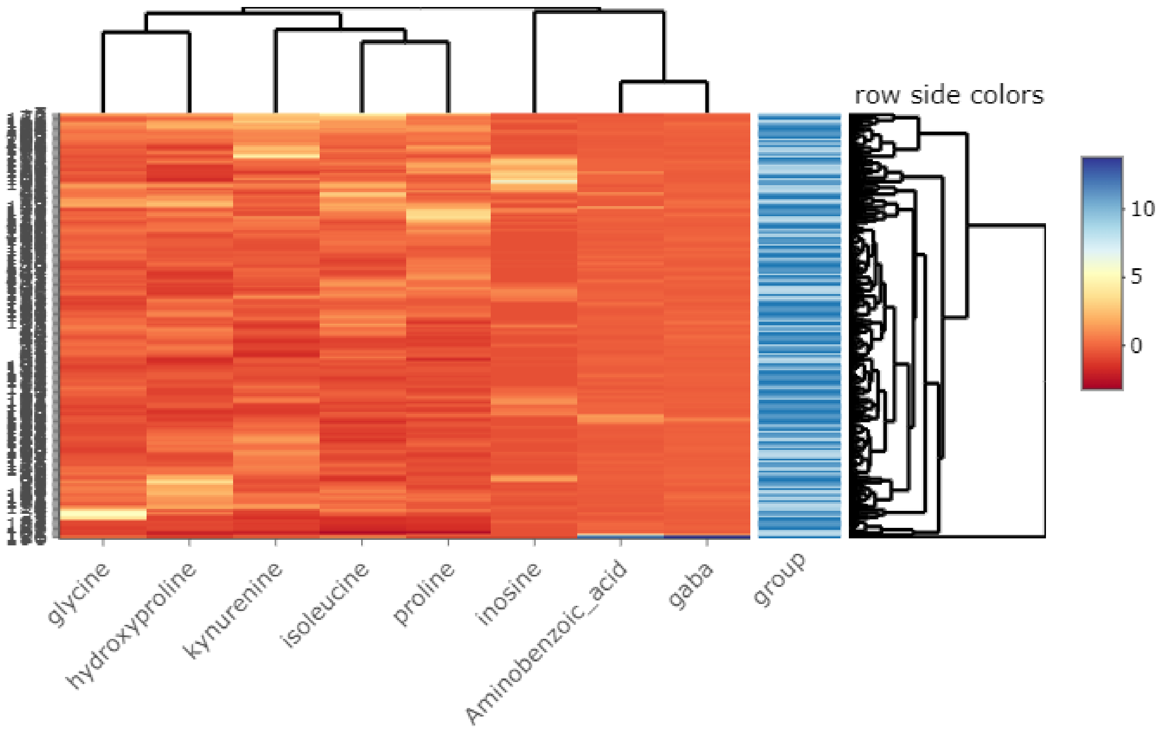

Supplementary Figure 8. Heatmap visualization of metabolites measured in QFT TB1 culture. Rows: samples; columns: metabolites. Light blue: HC; dark blue: TB+LTBI+NMP. Colour key indicates metabolite expression value: dark blue: lowest; dark red: highest.

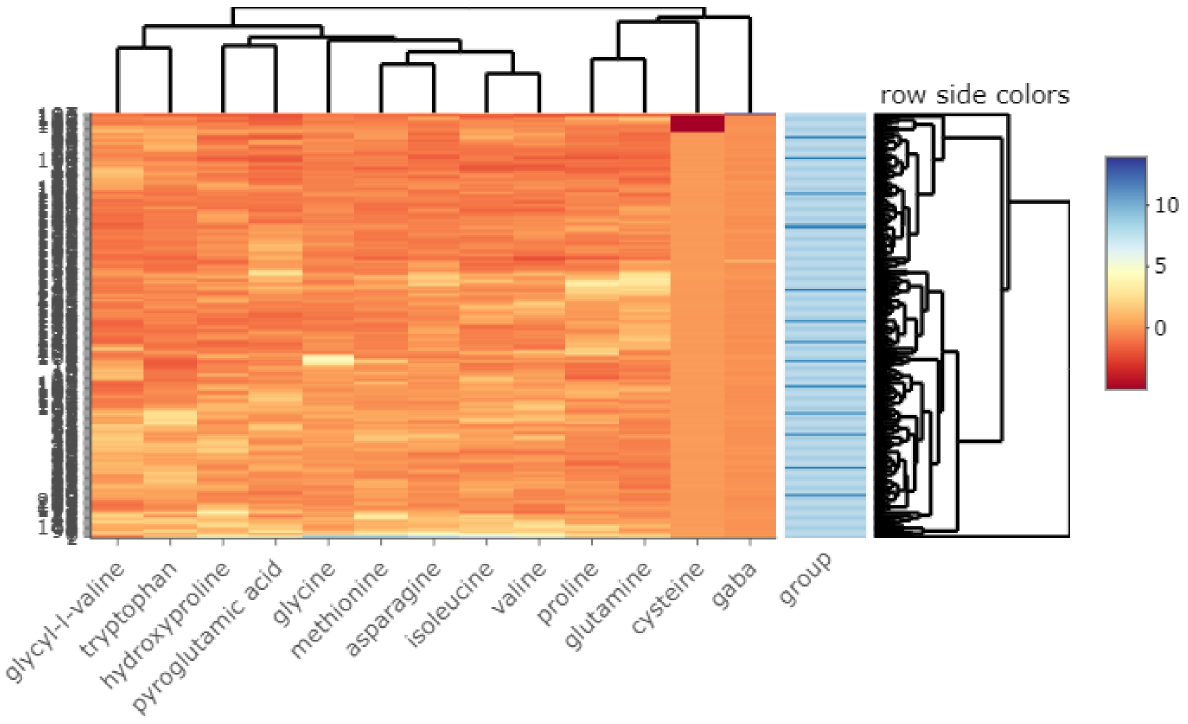

Supplementary Figure 9. Heatmap visualization of metabolites measured in QFT TB2 culture. Rows: samples; columns: metabolites. Light blue: TB; dark blue: HC+LTBI+NMP. Colour key indicates metabolite expression value: dark blue: lowest; dark red: highest.

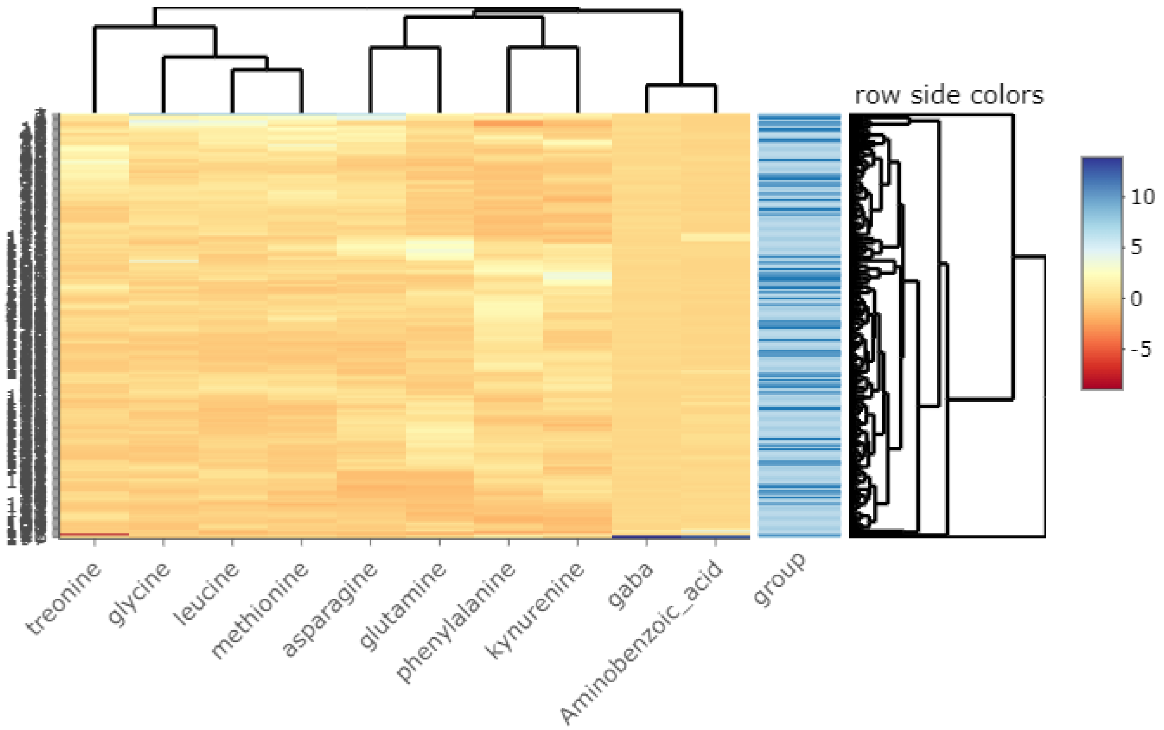

Supplementary Figure 10. Heatmap visualization of metabolites measured in QFT TB2 culture. Rows: samples; columns: metabolites. Light blue: TB+LTBI; dark blue: HC+NMP. Colour key indicates metabolite expression value: dark blue: lowest; dark red: highest.

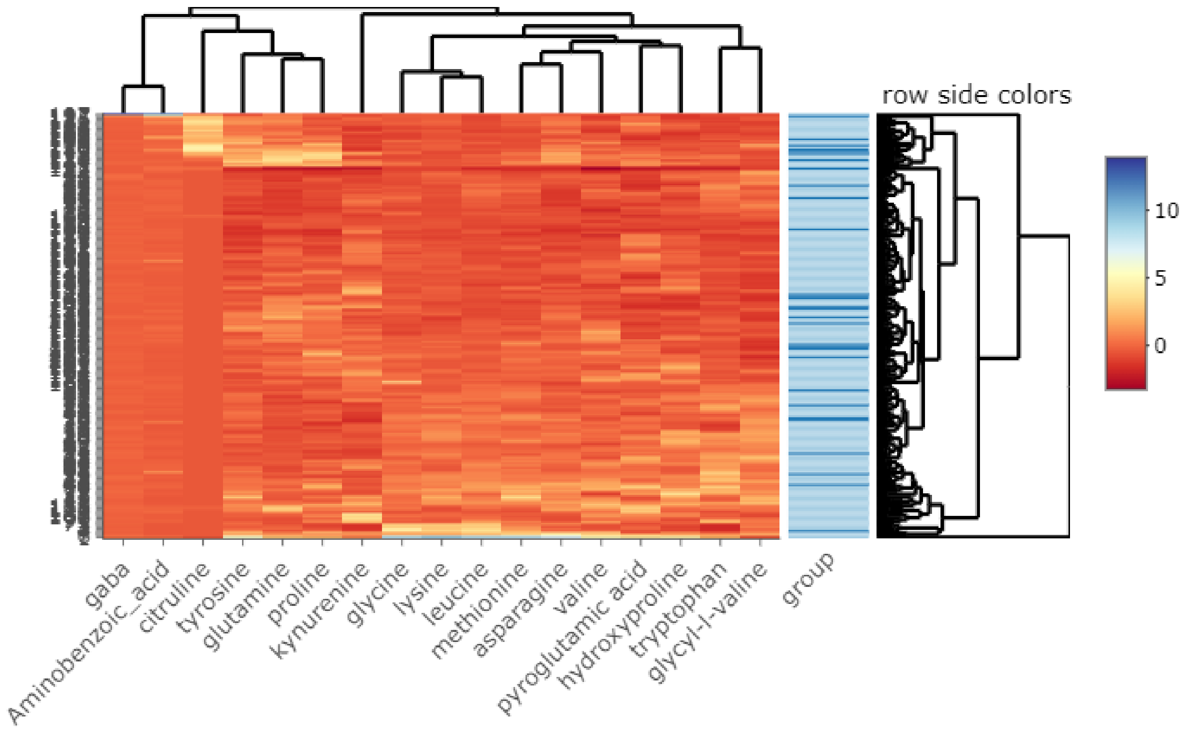

Supplementary Figure 11. Heatmap visualization of metabolites measured in QFT TB2 culture. Rows: samples; columns: metabolites. Light blue: TB+NMP; dark blue: HC+LTBI. Colour key indicates metabolite expression value: dark blue: lowest; dark red: highest.

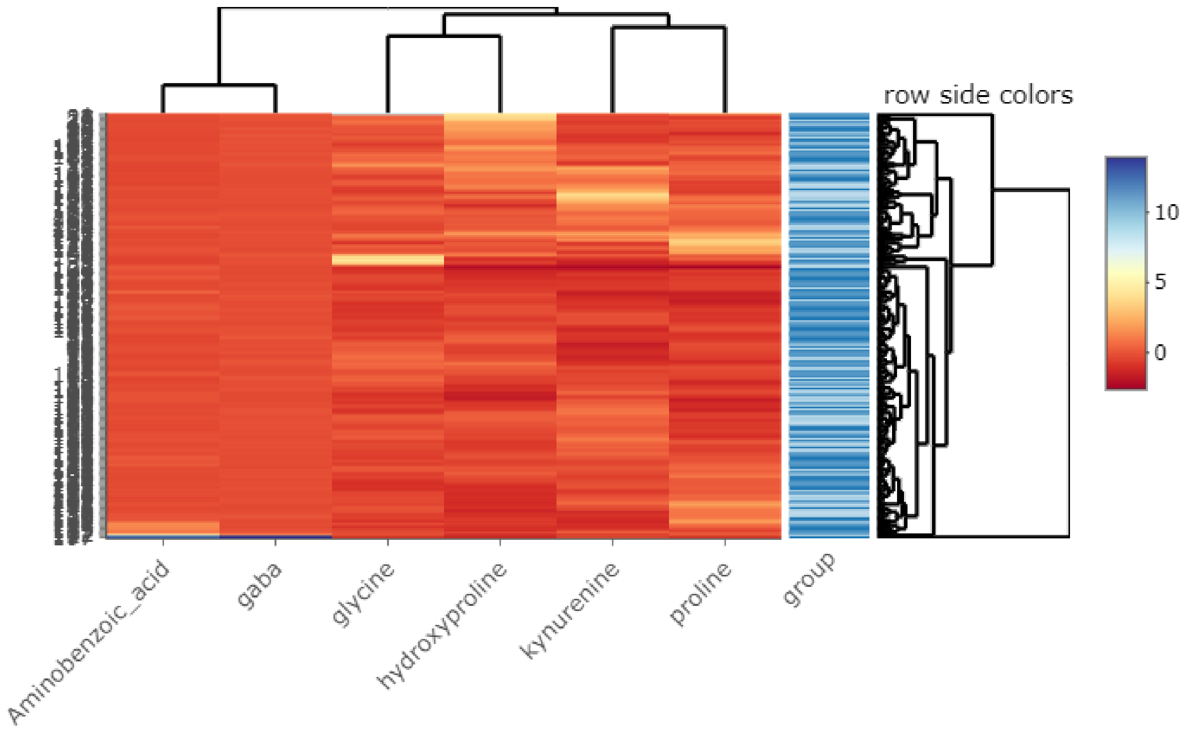

Supplementary Figure 12. Heatmap visualization of metabolites measured in QFT TB2 culture. Rows: samples; columns: metabolites. Light blue: HC; dark blue: TB+LTBI+NMP. Colour key indicates metabolite expression value: dark blue: lowest; dark red: highest.

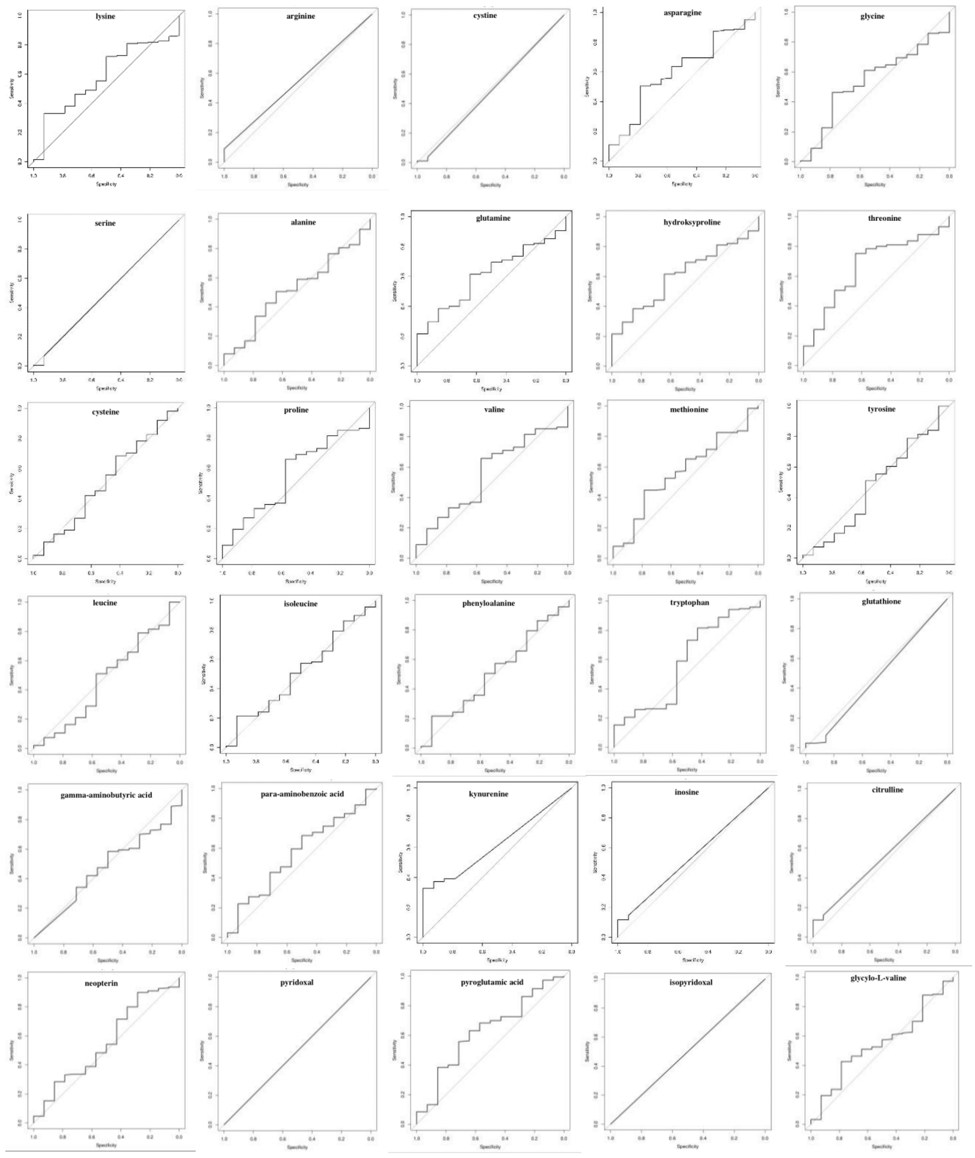

Supplementary Figure 13. ROC curves for the metabolites measured in serum for the differentiation of TB patients from other groups.

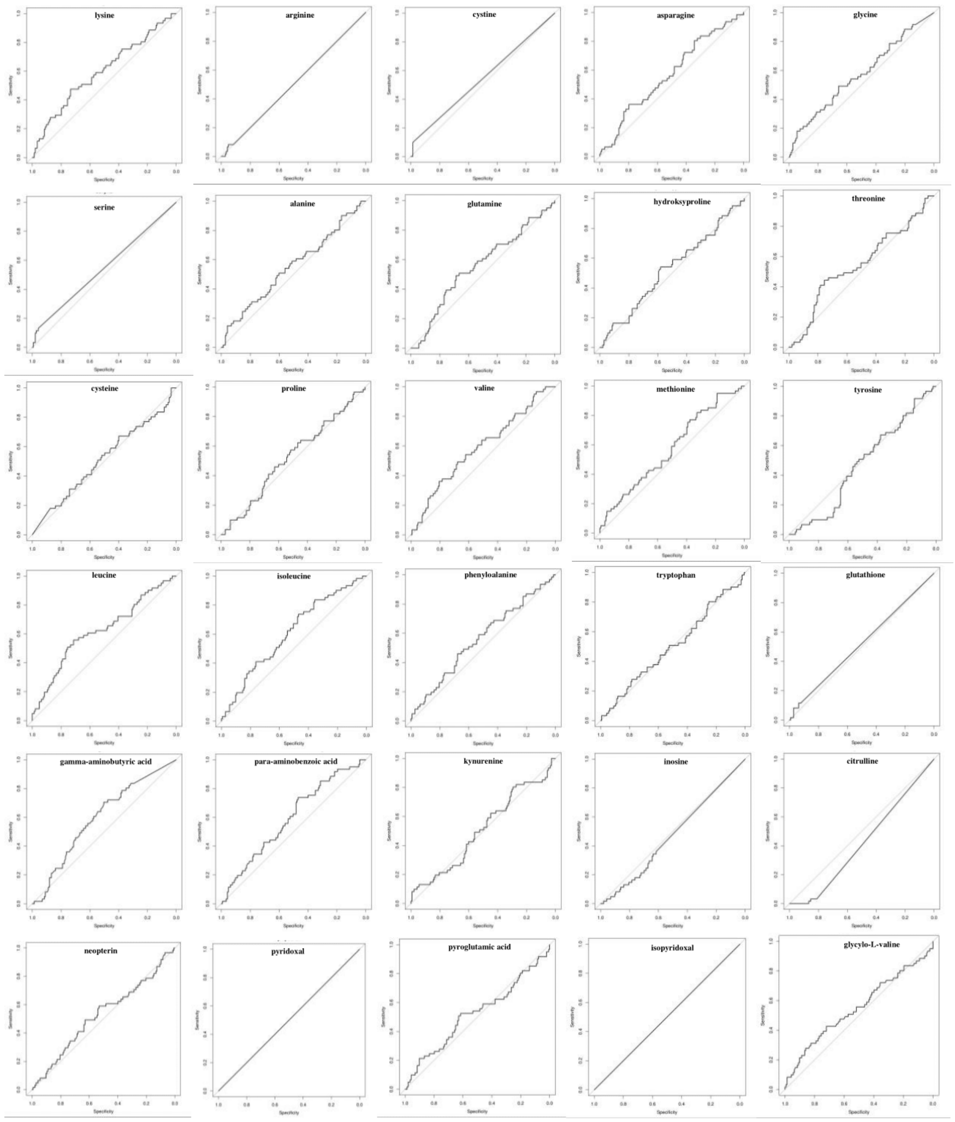

Supplementary Figure 14. ROC curves for the metabolites measured in serum for the differentiation of *M.tb*-infected individuals (TB+LTBI) from other groups (HC+NMP).

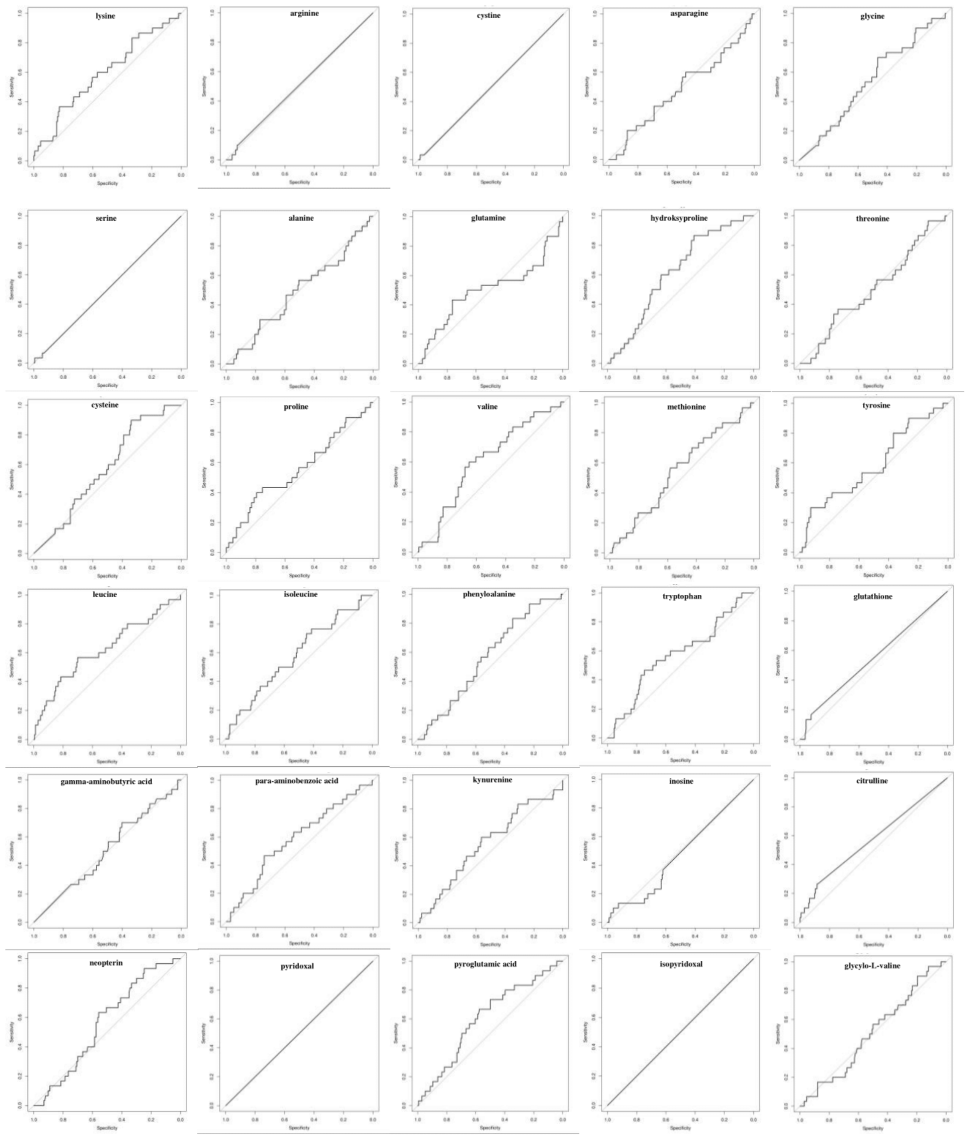

Supplementary Figure 15. ROC curves for the metabolites measured in serum for the differentiation of patients with pneumonia (TB+NMP) from other groups (HC+LTBI).

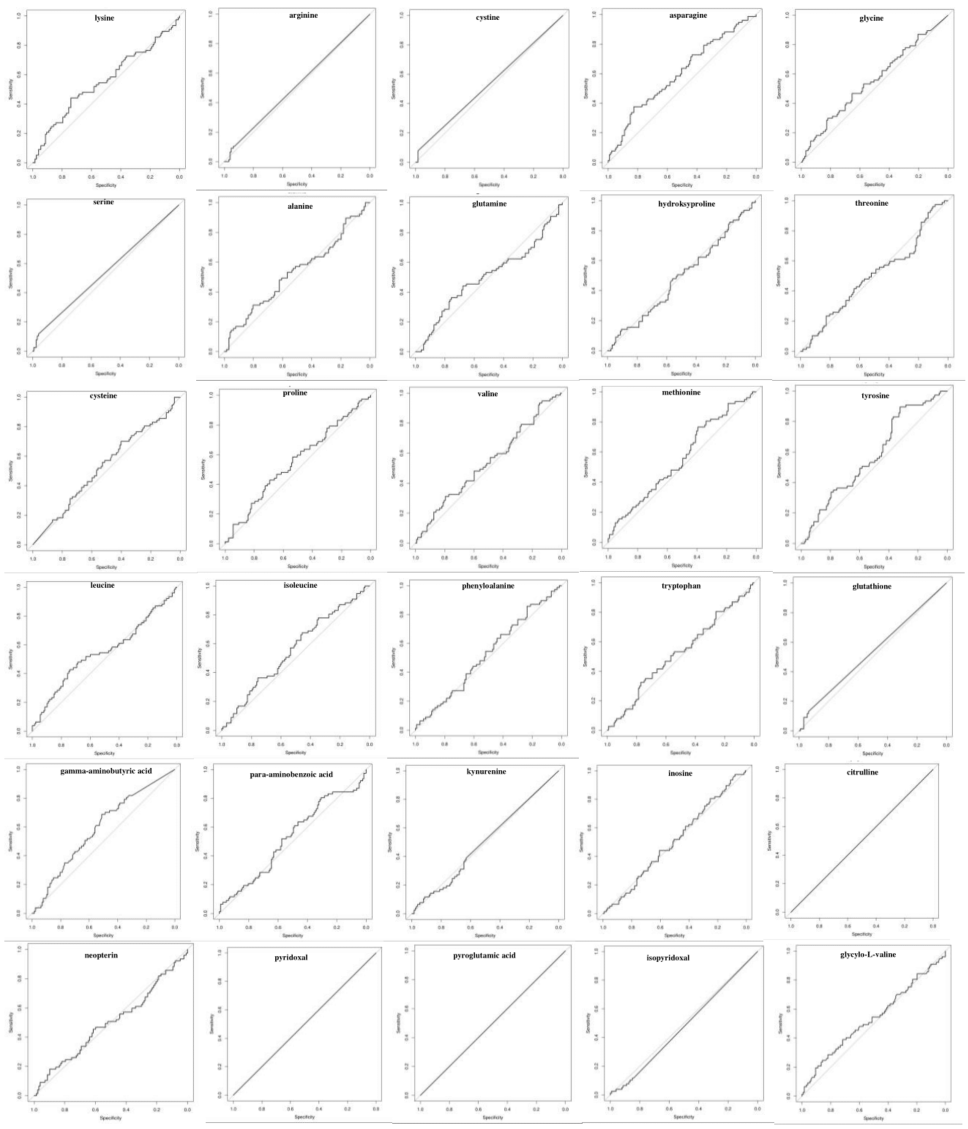

Supplementary Figure 16. ROC curves for the metabolites measured in serum for the differentiation of healthy controls (HC) from other groups (TB+LTBI+NMP).

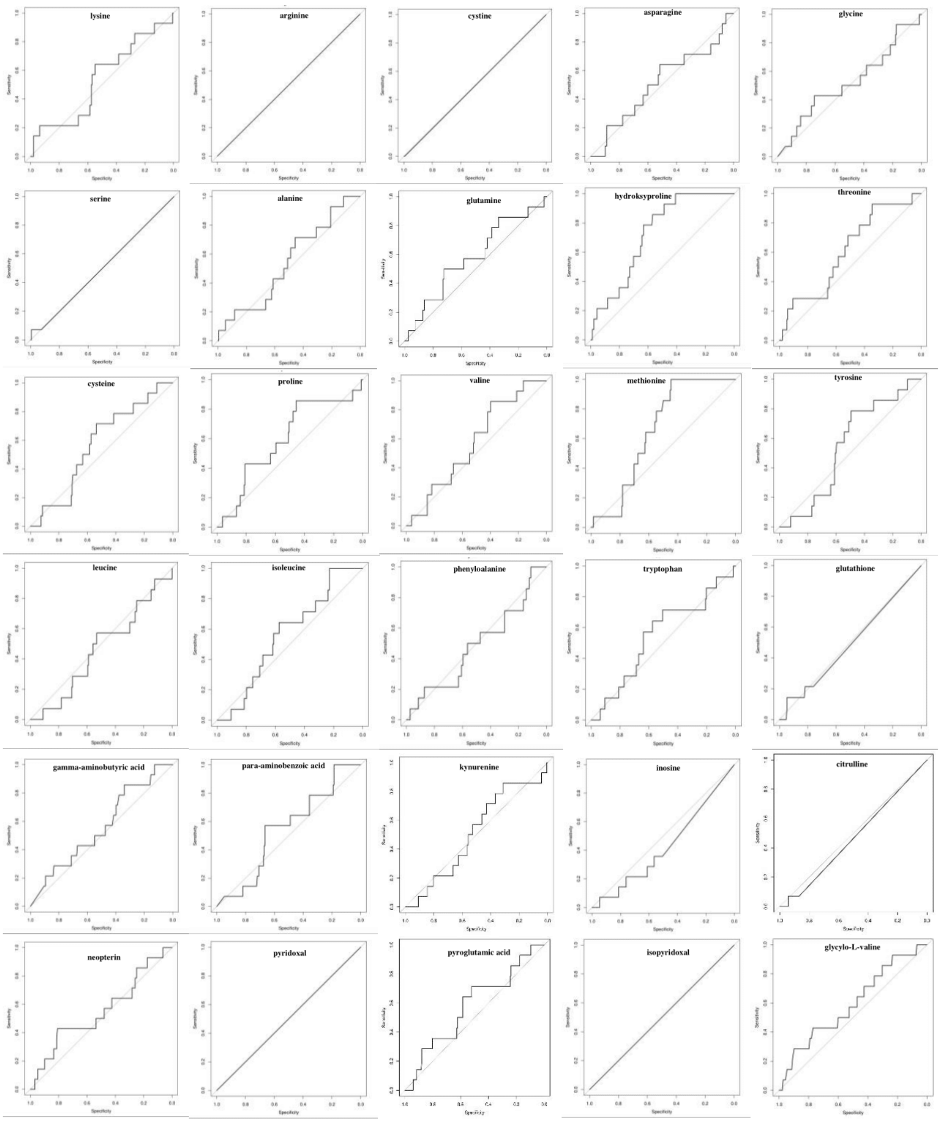

Supplementary Figure 17. ROC curves for the metabolites measured in OFT TB1 cultures for the differentiation of TB patients from other groups.

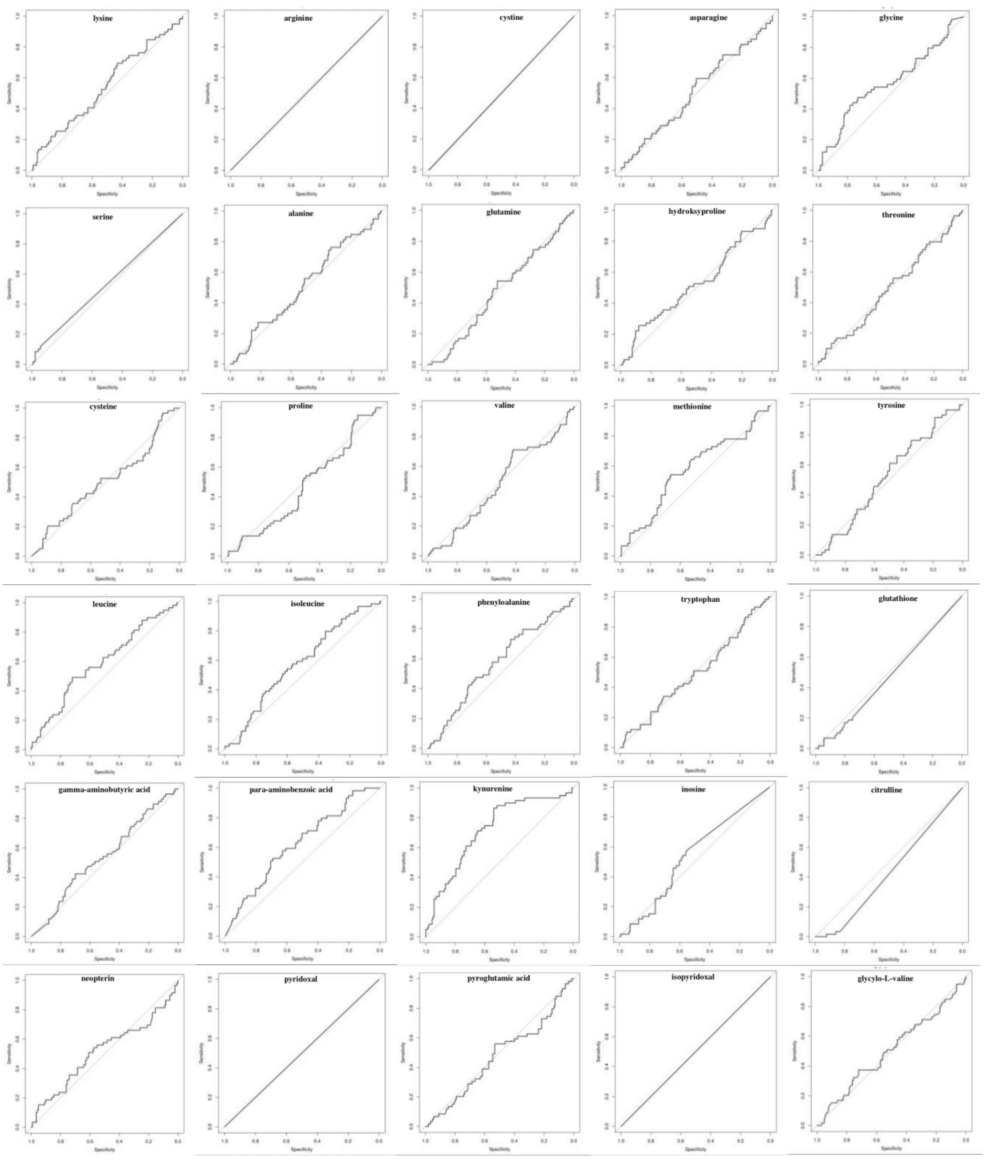

Supplementary Figure 18. ROC curves for the metabolites measured in OFT TB1 cultures for the differentiation of *M.tb*-infected individuals (TB+LTBI) from other groups (HC+NMP).

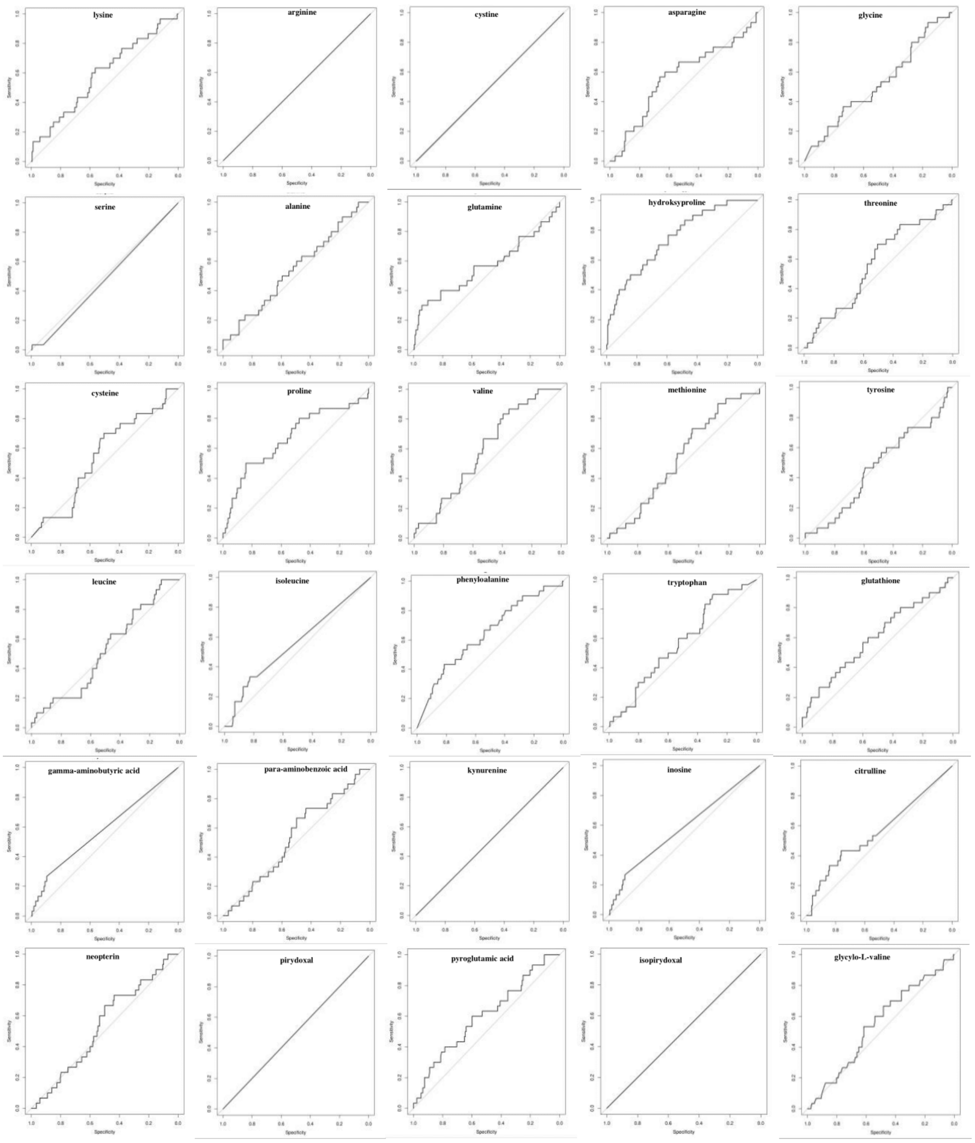

Supplementary Figure 19. ROC curves for the metabolites measured in OFT TB1 cultures for the differentiation of patients with pneumonia (TB+NMP) from other groups (HC+LTBI)

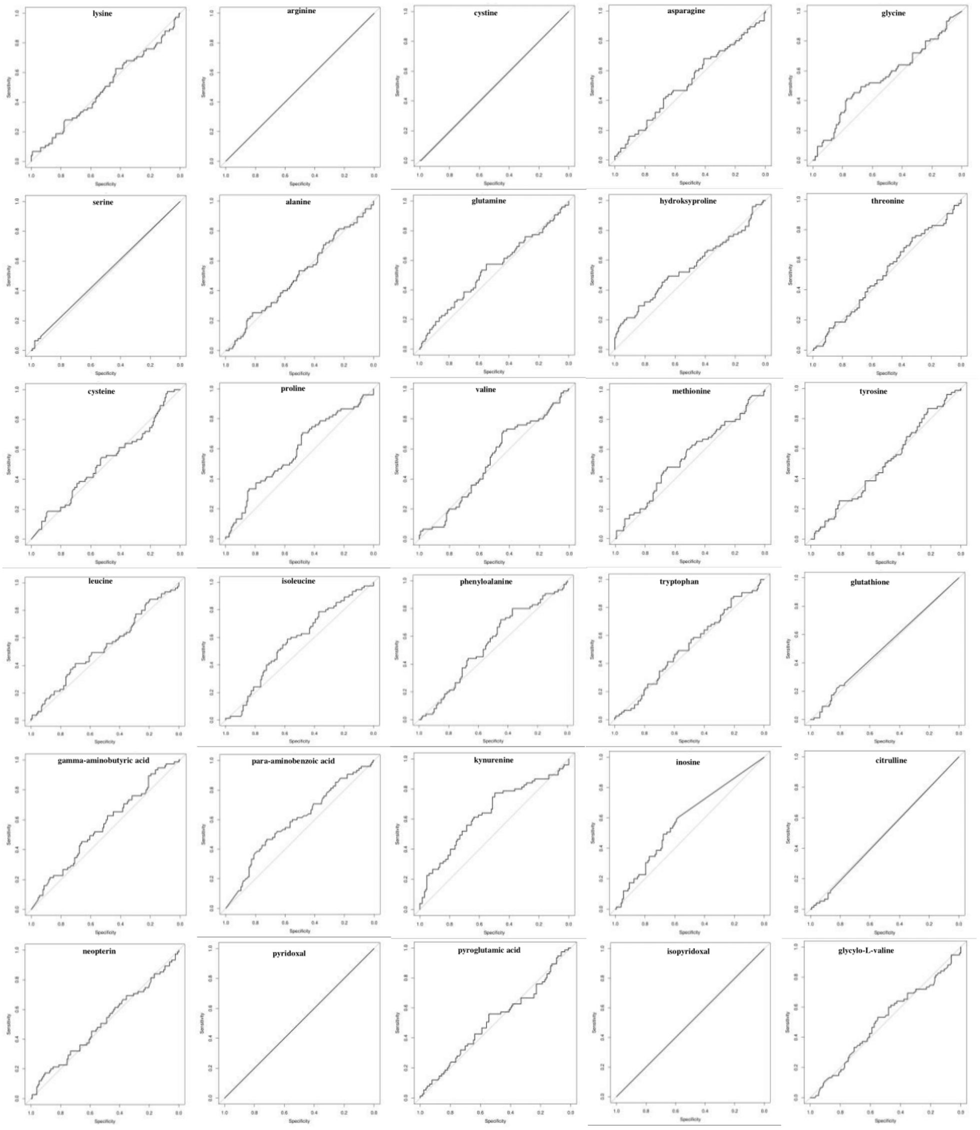

Supplementary Figure 20. ROC curves for the metabolites measured in OFT TB1 cultures for the differentiation of healthy controls (HC) from other groups (TB+LTBI+NMP).

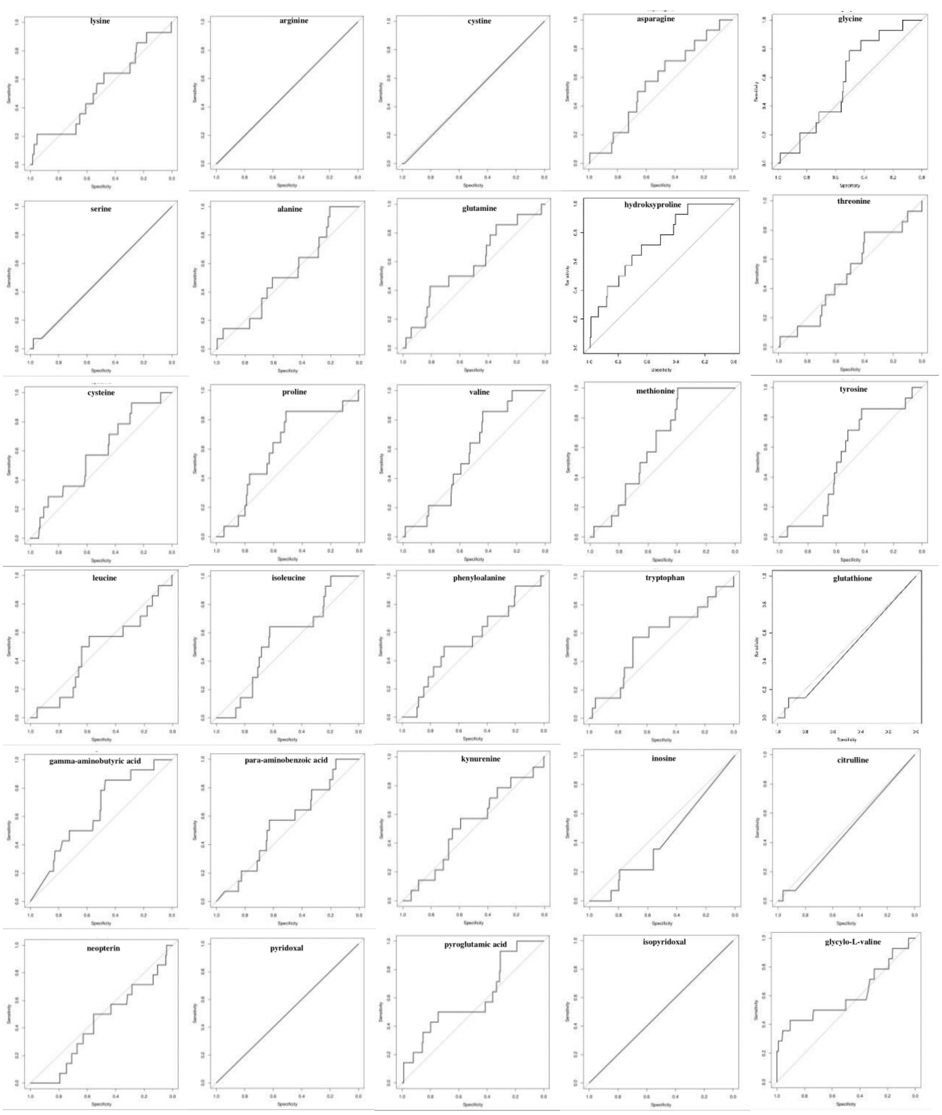

Supplementary Figure 21. ROC curves for the metabolites measured in OFT TB2 cultures for the differentiation of TB patients from other groups.

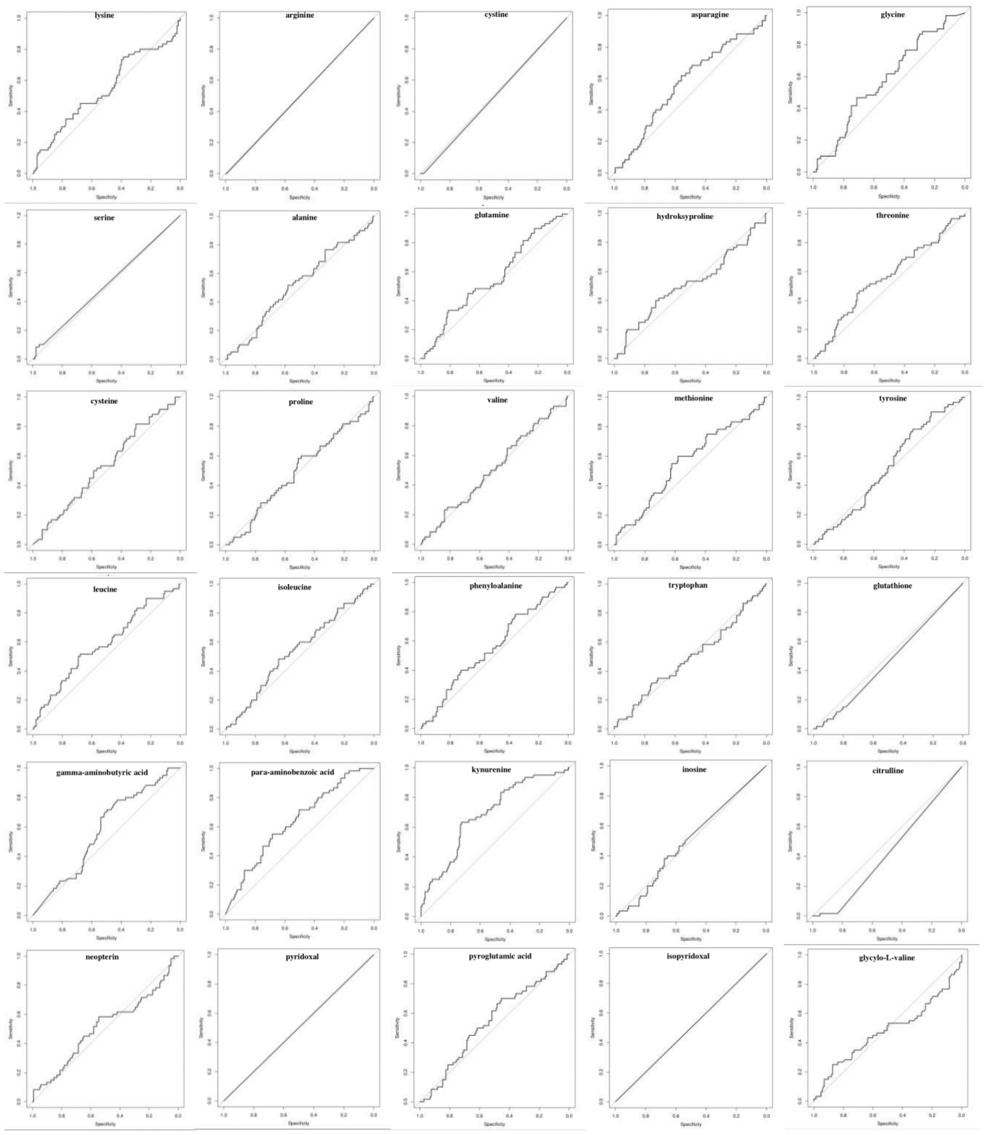

Supplementary Figure 22. ROC curves for the metabolites measured in OFT TB2 cultures for the differentiation of *M.tb*-infected individuals (TB+LTBI) from other groups (HC+NMP).

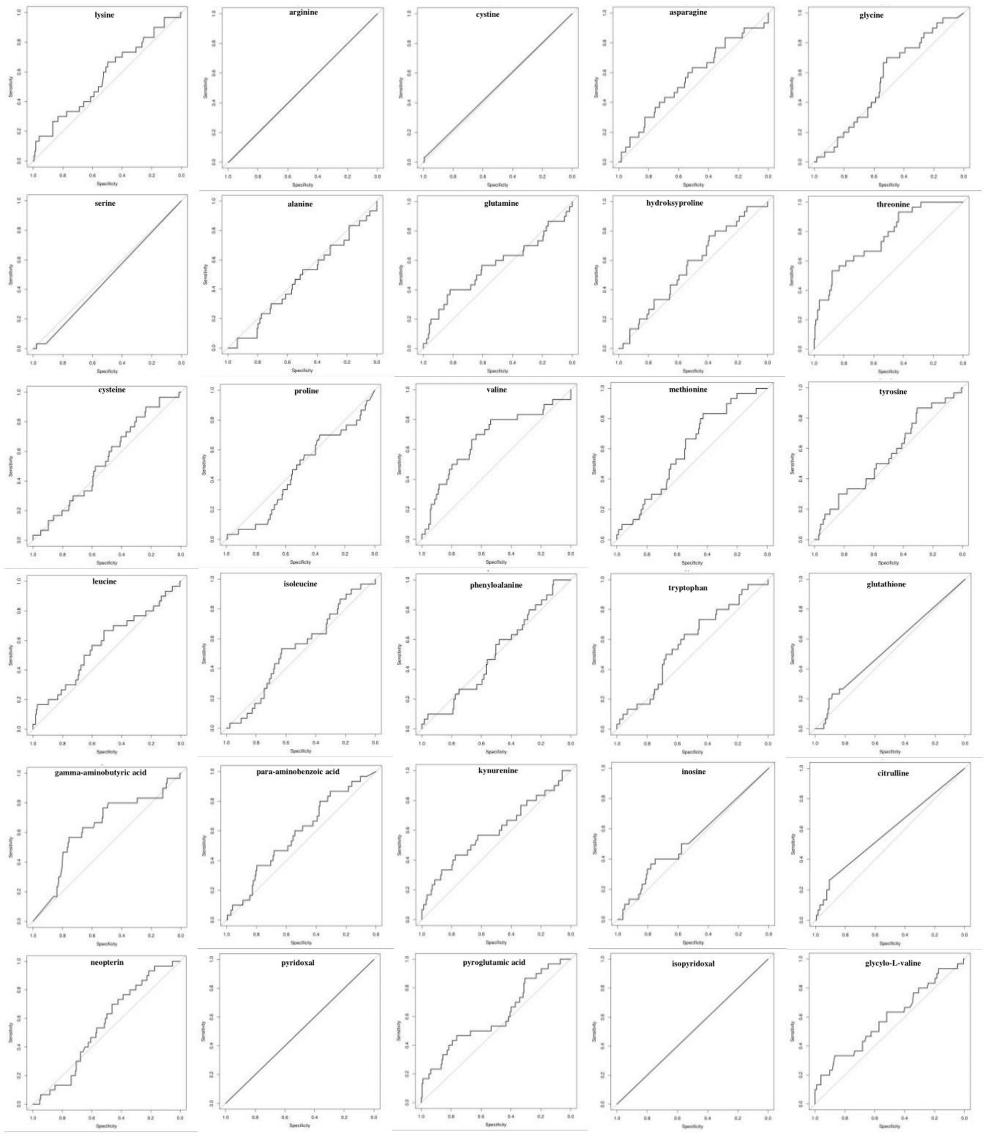

Supplementary Figure 23. ROC curves for the metabolites measured in OFT TB2 cultures for the differentiation of patients with pneumonia (TB+NMP) from other groups (HC+LTBI)

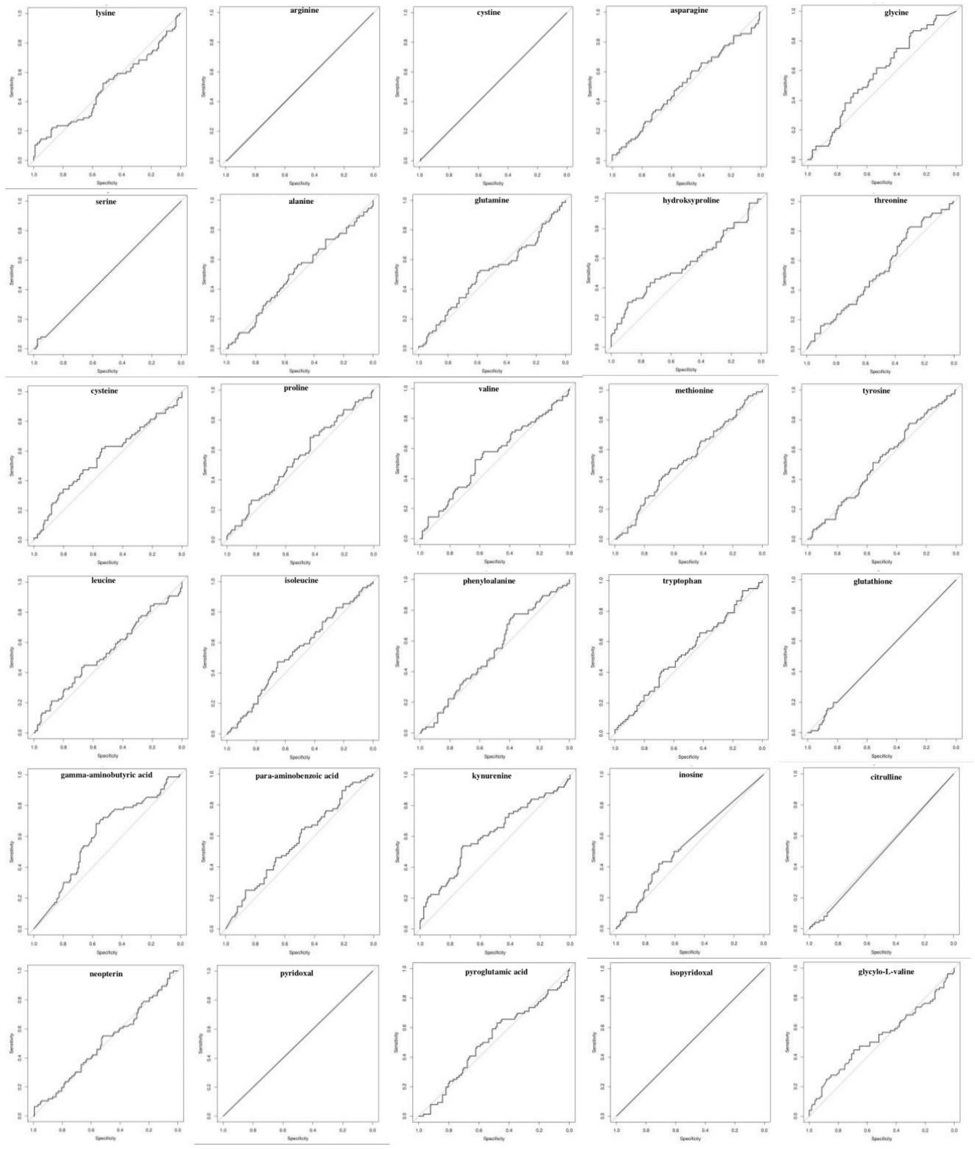

Supplementary Figure 24. ROC curves for the metabolites measured in OFT TB2 cultures for the differentiation of healthy controls (HC) from other groups (TB+LTBI+NMP).

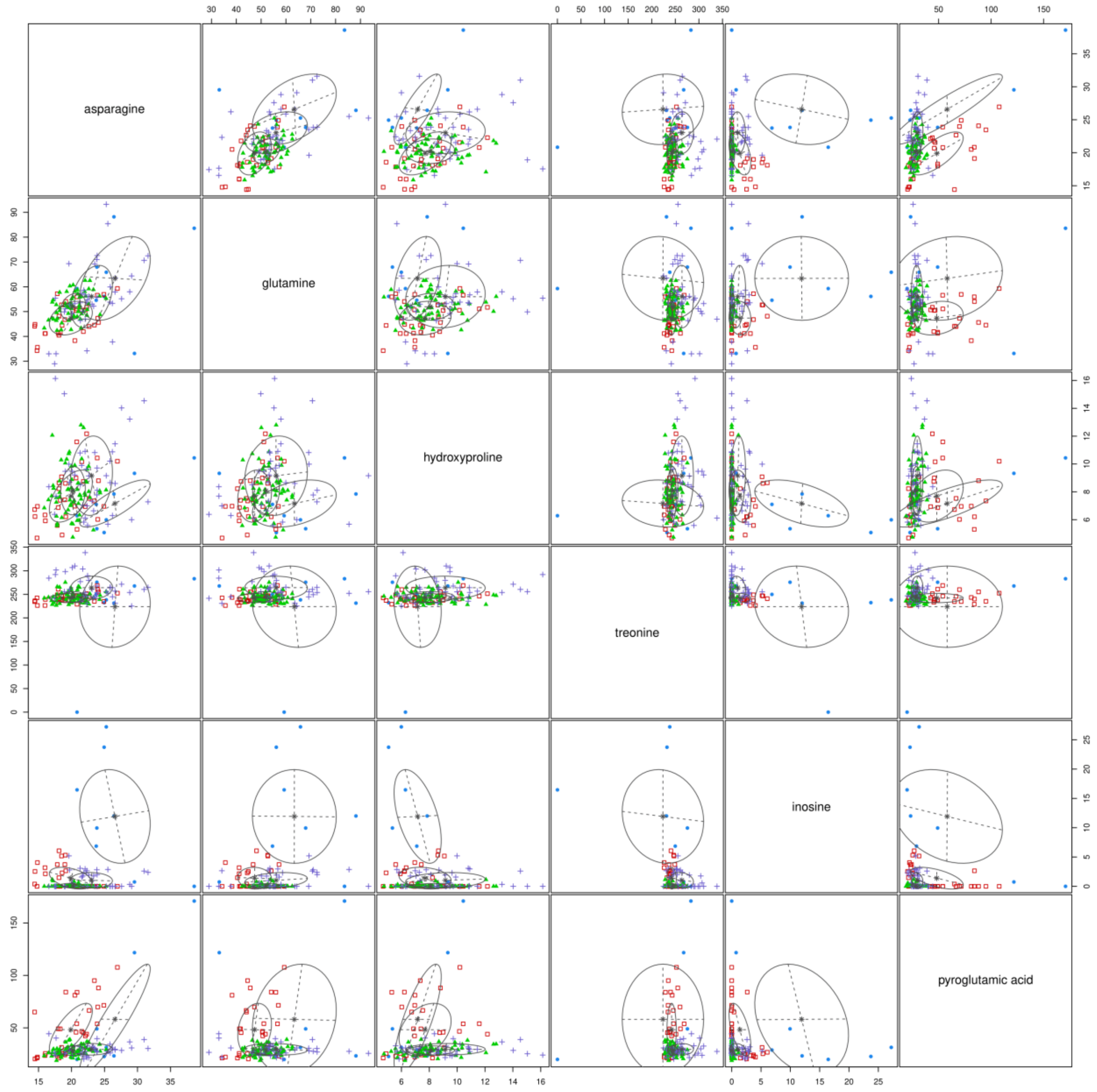

Supplementary Figure 25. Unsupervised model-based clustering results of metabolites measured in serum of TB patients vs HC subjects

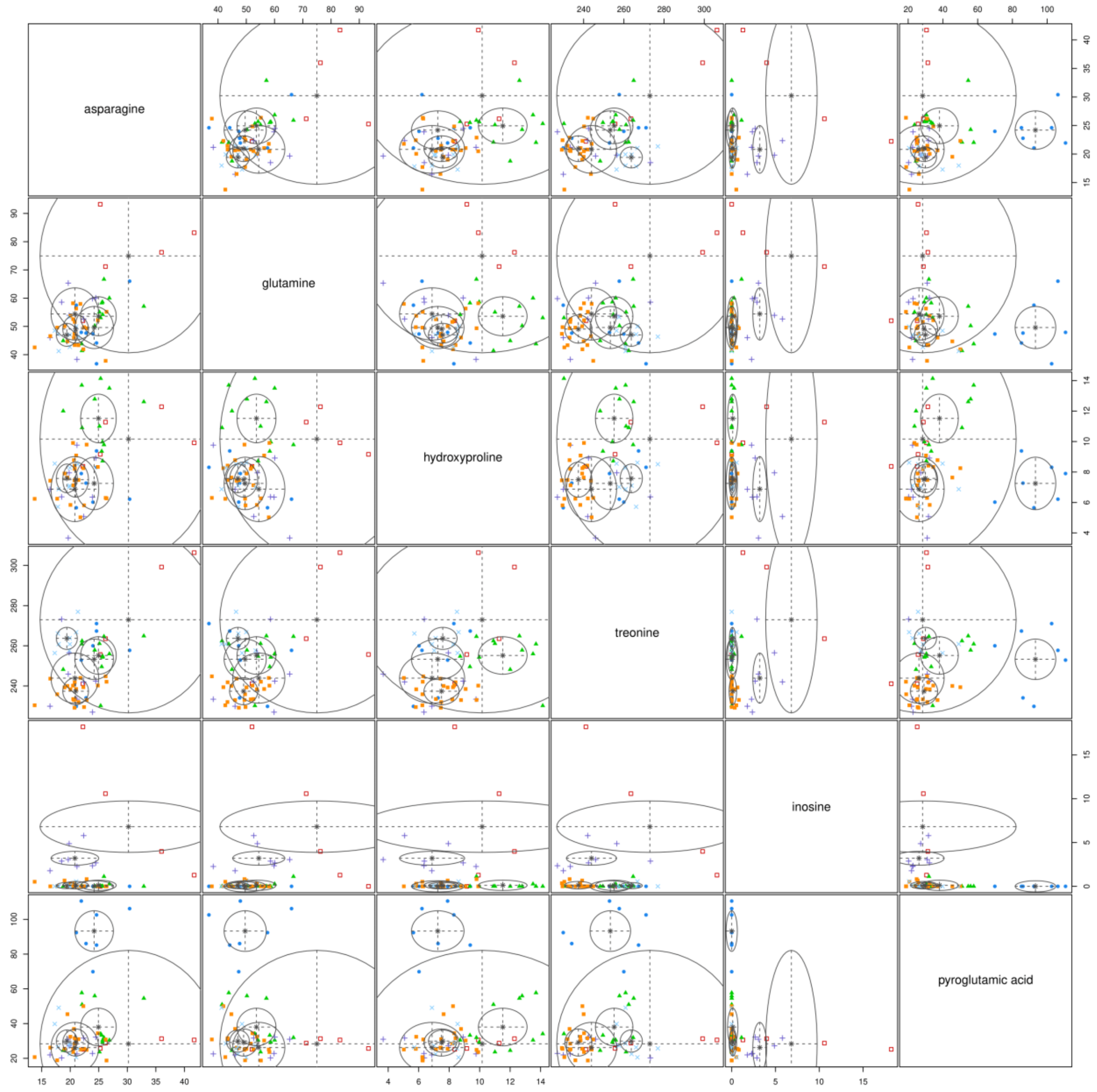

Supplementary Figure 26. Unsupervised model-based clustering results of metabolites measured in serum of TB patients vs LTBI subjects

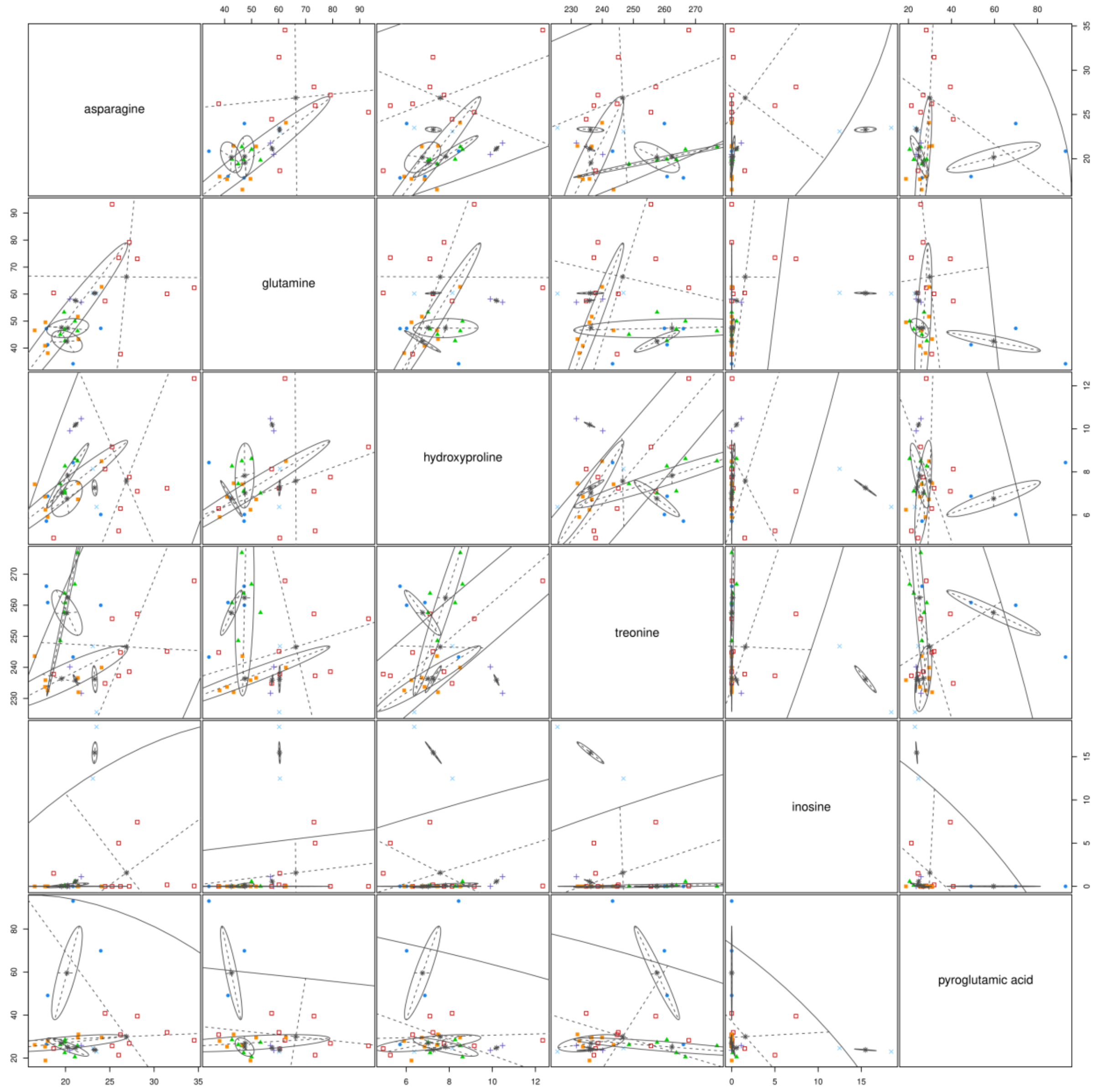

Supplementary Figure 27. Unsupervised model-based clustering results of metabolites measured in serum of TB patients vs NMP patients

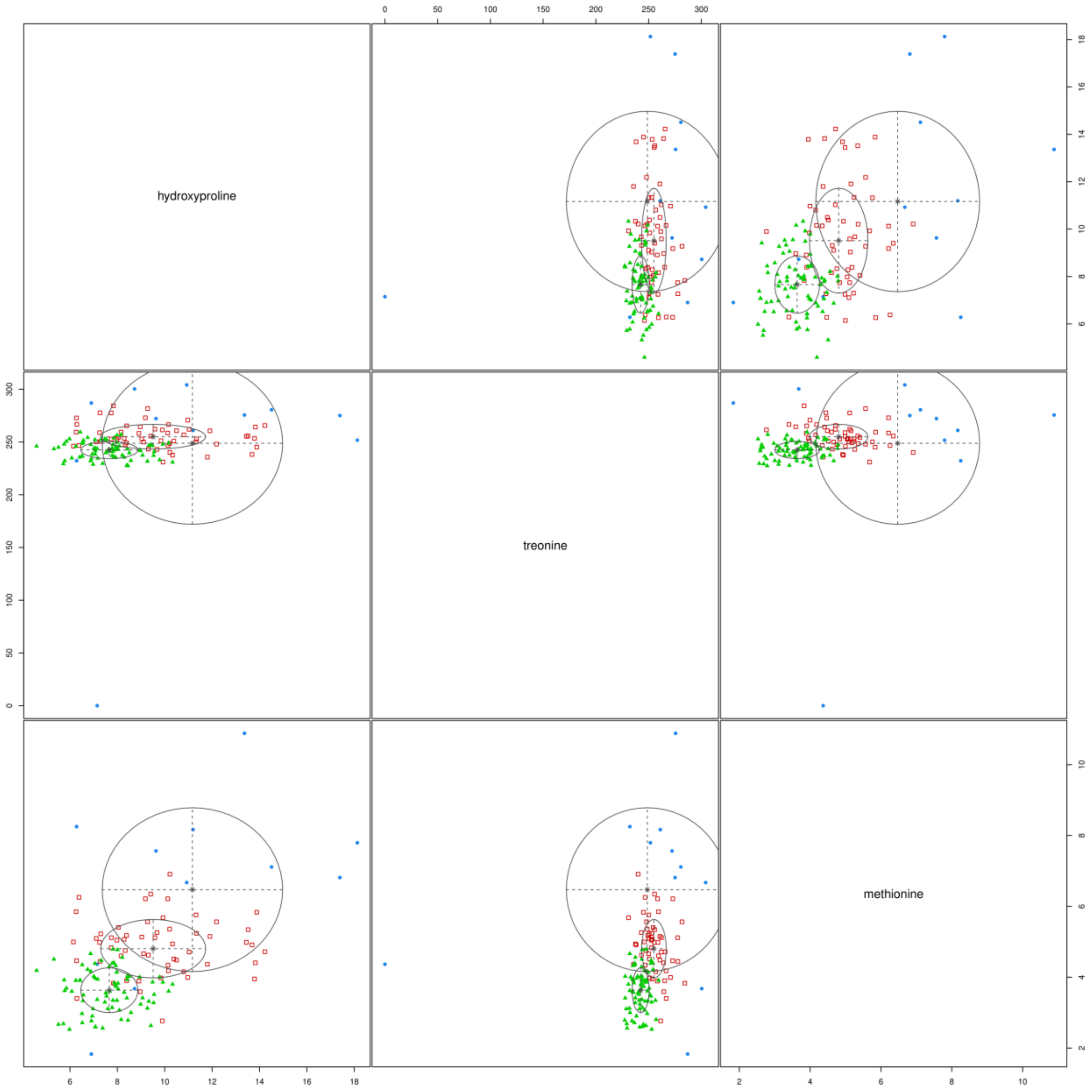

Supplementary Figure 28. Unsupervised model-based clustering results of metabolites measured in OFT TB1 cultures of TB patients vs HC subjects

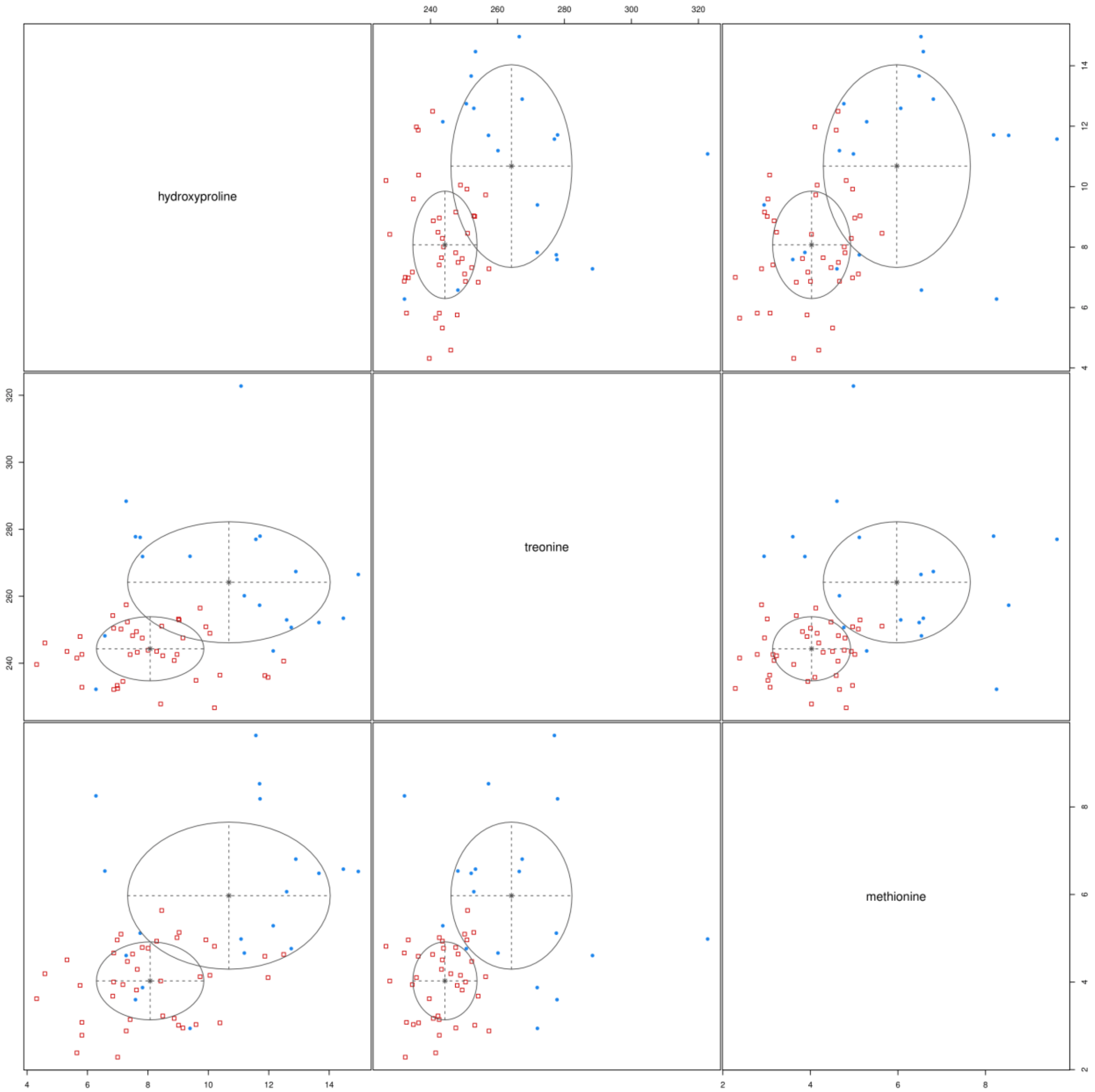

Supplementary Figure 29. Unsupervised model-based clustering results of metabolites measured in OFT TB1 cultures of TB patients vs LTBI subjects

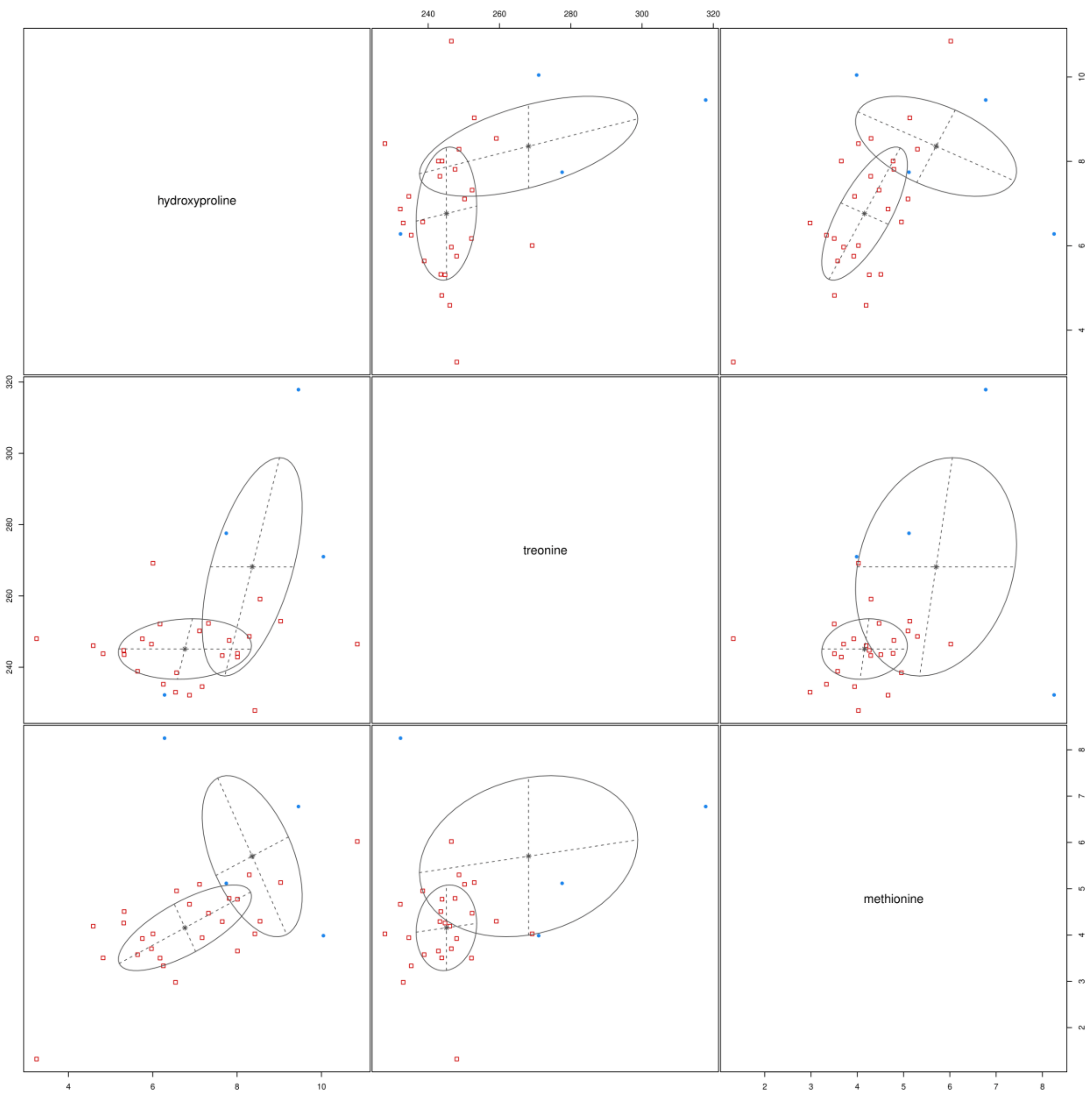

Supplementary Figure 30. Unsupervised model-based clustering results of metabolites measured in OFT TB1 cultures of TB patients vs NMP patients

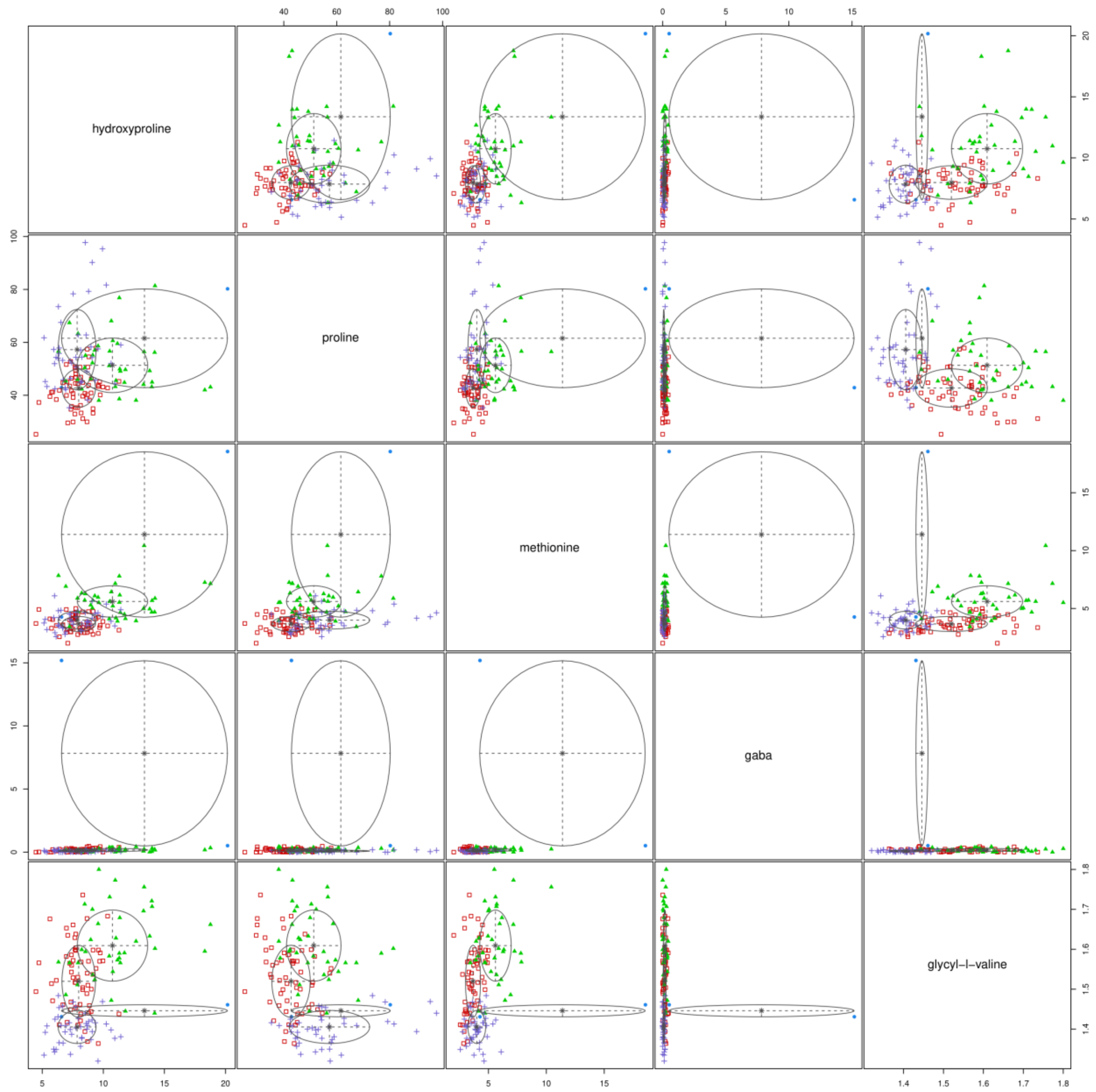

Supplementary Figure 31. Unsupervised model-based clustering results of metabolites measured in OFT TB2 cultures of TB patients vs HC subjects

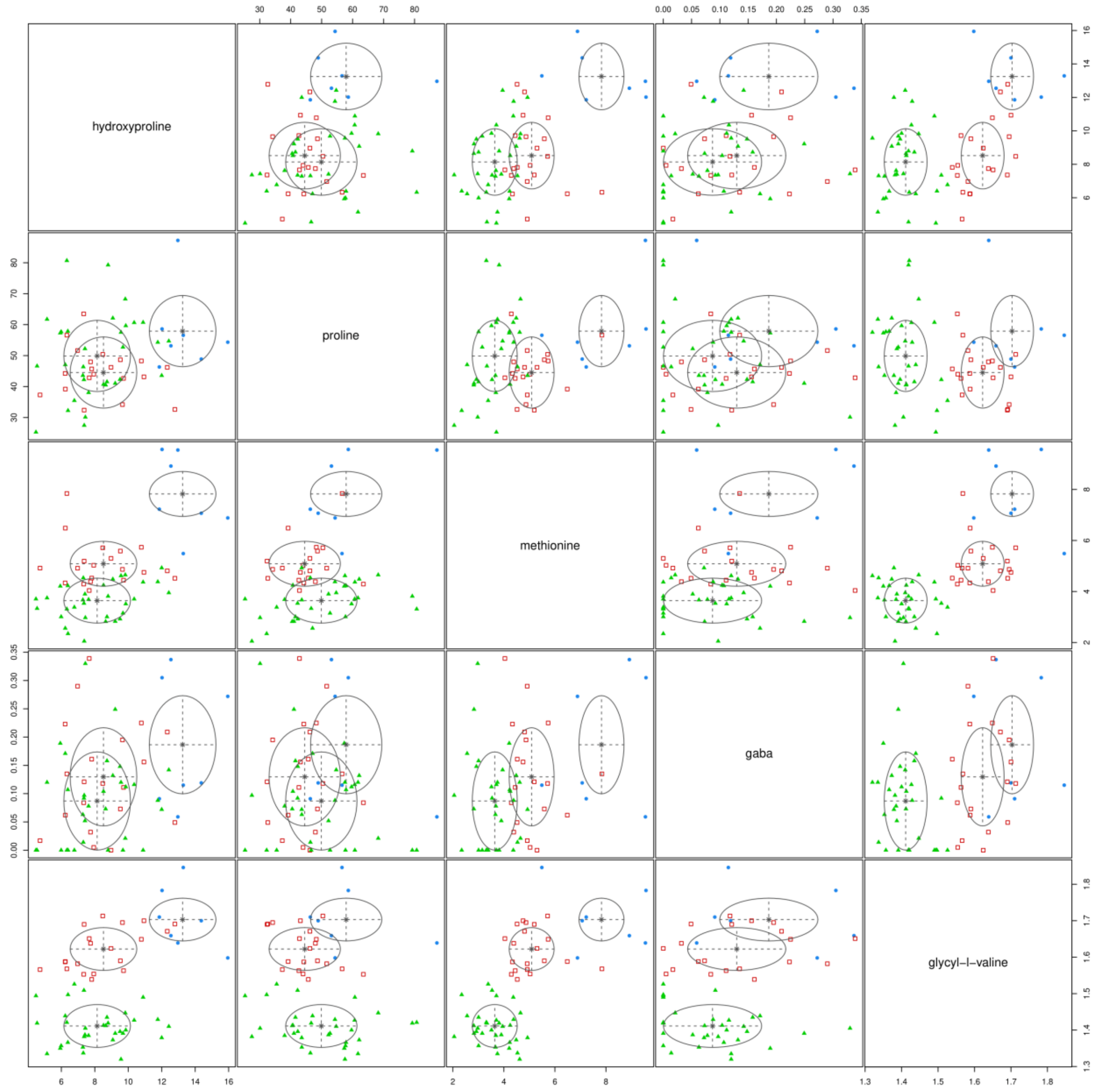

Supplementary Figure 32. Unsupervised model-based clustering results of metabolites measured in OFT TB1 cultures of TB patients vs LTBI subjects

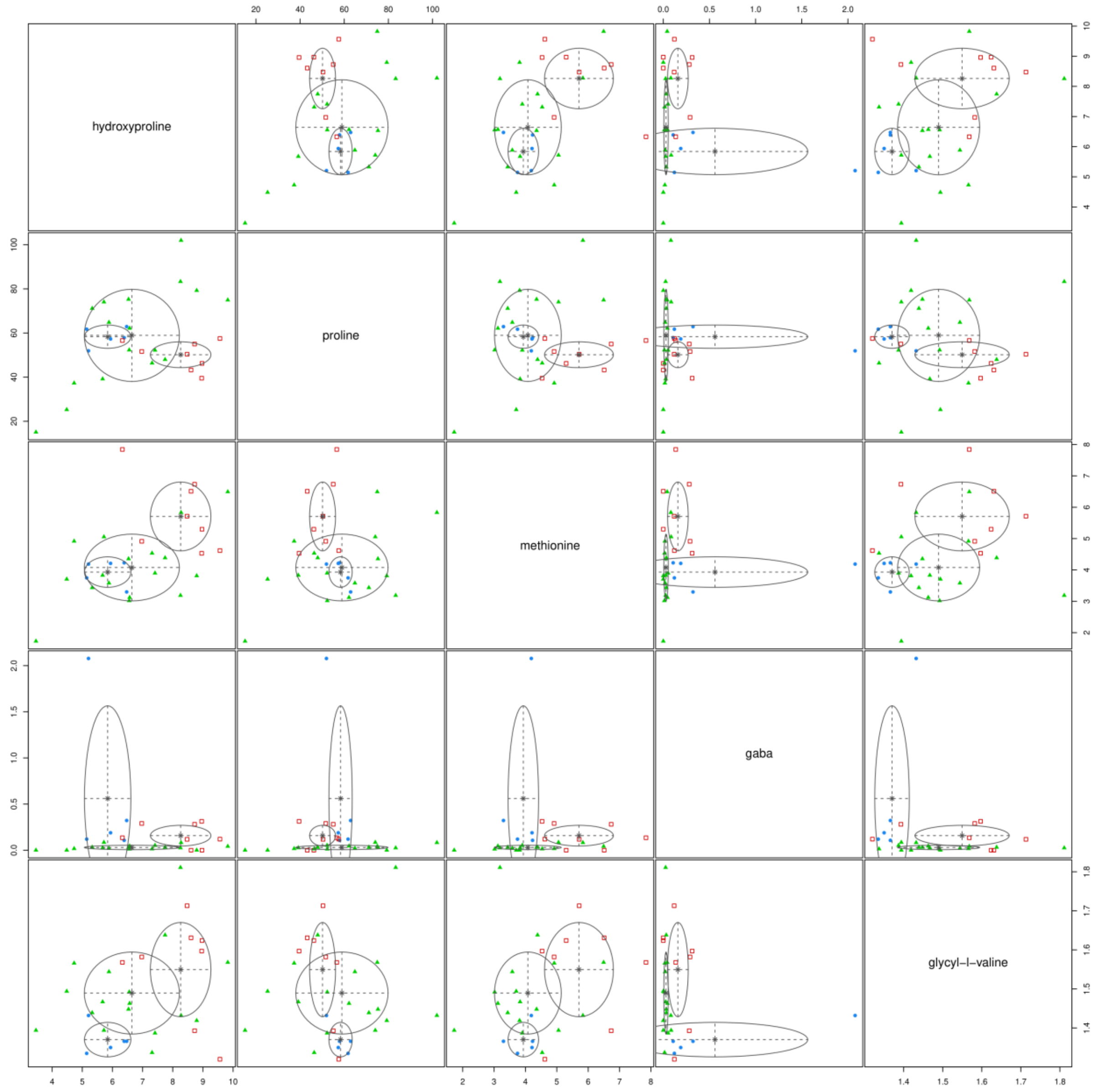

Supplementary Figure 33. Unsupervised model-based clustering results of metabolites measured in OFT TB1 cultures of TB patients vs NMP patients

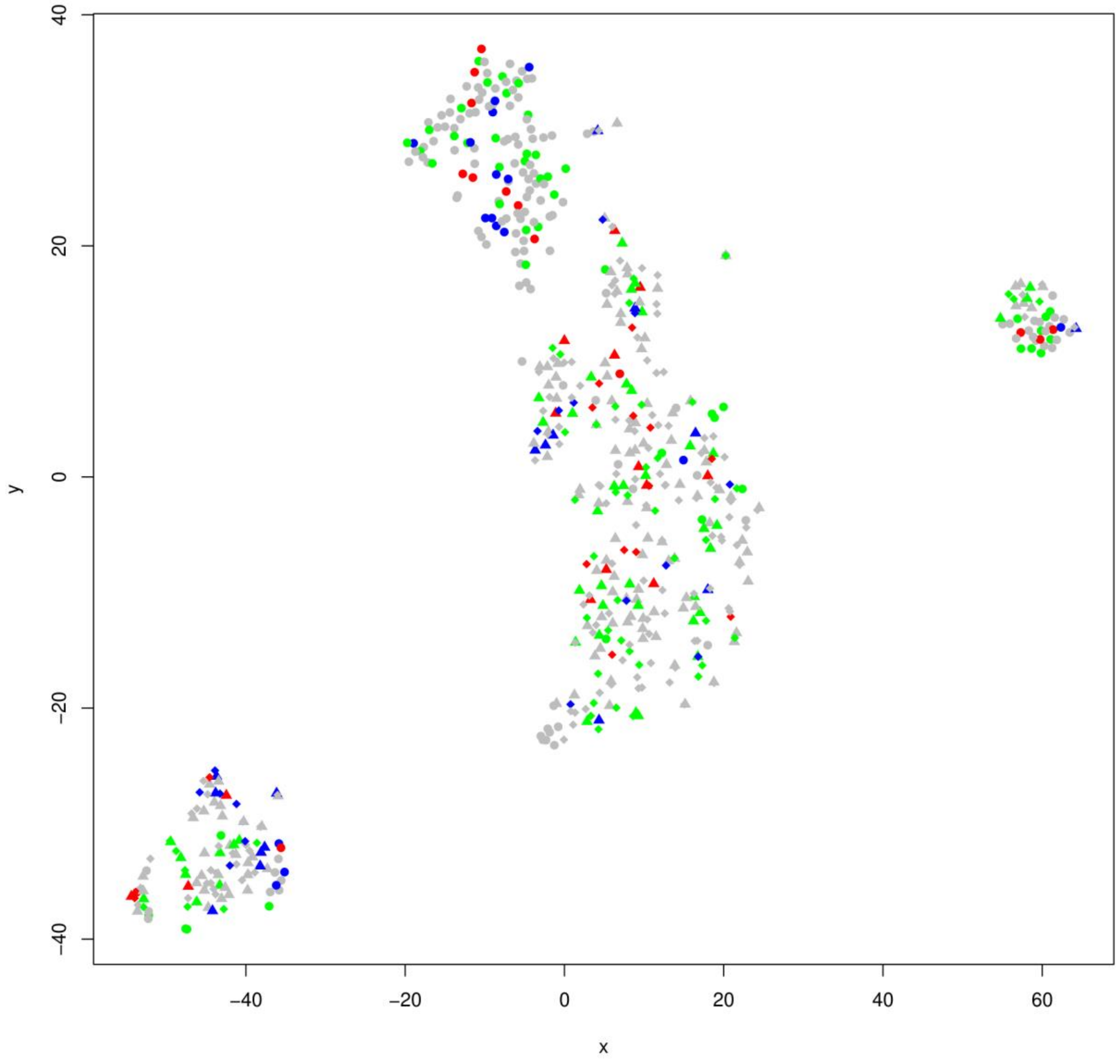

Supplementary Figure 34. Dimension reduction plot by means ogf the t-SNE method with the aid of the Rtsne package in R. We used the exact t-SNE algorithm with perplexity equal to 30. In the plot, the circles represent “serum”, triangles “QFT TB1” and rectangles “QFT TB2”. As far as the color coding is concerned: grey represents HC, green LTBI, blue NMP and red TB.
