# Supplementary material for: Targeted metabolomics analysis of serum and Mycobacterium tuberculosis antigen-stimulated blood cultures of pediatric patients with active and latent tuberculosis
Source: Sci Rep. 2022 Mar 8;12:4131. doi: 10.1038/s41598-022-08201-4 (PMC8904507; doi:10.1038/s41598-022-08201-4)
Supplement: Supplementary file 2 — Supplementary Information 2. [file 41598_2022_8201_MOESM2_ESM.pdf]

Supplementary Tables

Targeted metabolomics analysis of serum and *Mycobacterium tuberculosis* antigens-stimulated blood cultures of pediatric patients with active and latent tuberculosis

Druszczynska Magdalena[1], Seweryn Michal[2], Sieczkowska Marta[3], Kowalewska-Pietrzak Magdalena[4], Pankowska Anna[4], Godkowicz Magdalena[1], Szewczyk Rafał[3]

Supplementary Table S1. Mean (M), median (Me) and standard deviation (SD) of metabolites measured in serum in each group, together with the p-value (uncorrected) of the Kruskal-Wallis test for equality of location parameter across study groups

Supplementary Table S2. Mean (M), median (Me) and standard deviation (SD) of metabolites measured in M.tb-stimulated whole blood QFT cultures in each group, together with the p-value (uncorrected) of the Kruskal-Wallis test for equality of location parameter across study groups

Supplementary Table S3. Ion source parameters.

Supplementary Table S4. Compound-dependent parameters in the targeted scheduled multiple reaction monitoring (sMRM) LC-MS/MS method

Supplementary Table S5. Linearity and working ranges of the/a quantitative LC-MS/MS method.

Supplementary Table S1. Mean (M), median (Me) and standard deviation (SD) of metabolites measured in serum in each group, together with the p-value (uncorrected) of the Kruskal-Wallis test for equality of location parameter across study groups

Metabolite	TB	LTBI	NMP	HC	p-value
	M; Me; SD	M; Me; SD	M; Me; SD	M; Me; SD	
lysine (Lys)	25.6; 23.4; 5.5	23.5; 21.4; 5.6	21.1; 21.4; 4.8	24.1; 23.4; 4.8	0.083
arginine (Arg)	0.001; 0.001; 0.003	0; 0; 0.002	0; 0; 0	0.05; 0; 0.5	0.335
cystine	0.02; 0.001; 0.08	0.01; 0; 0.04	0.02; 0; 0.07	0.01; 0.001; 0.04	0.506
asparagine (Asn)	24.5; 23.4; 6.1	22.2; 21.4; 3.6	23.4; 23.4; 3.8	23.1; 23.4; 3.6	0.151
glycine (Gly)	26.7; 19.6; 23.3	26.7; 26.1; 15.8	21.7; 23.4; 8.7	21.2; 19.6; 13.8	0.120
serine (Ser)	1.8; 0.001; 6.9	1.4; 0.001; 5.9	0; 0; 0	1.1; 0.001; 6.7	0.046
alanine (Ala)	90.8; 83.6; 18.6	91.3; 83.6; 20.7	83.8; 83.6; 22.0	89.2; 83.6; 22.3	0.417
glutamine (Gln)	42.6; 38.8; 9.2	44.1; 44,3; 9.1	46.9; 46.9; 5.0	44.5; 44.3; 7.4	0.153
hydroxyproline (Hyp)	8.6; 8.5; 2.7	8.9; 8.5; 2.9	7.5; 6.3; 2.0	9.4; 8.5; 2.8	0.059
threonine (Thr)	342.4; 375.5; 84.1	325.1; 375.5; 96.6	274.1; 375.5; 118.7	328.0; 375.5; 93.8	0.170
cysteine (Cys)	91.2; 99.8; 55.5	104.0; 99.8; 38.0	112.5; 99.8; 22.0	112.2; 99.8; 30.5	0.504
proline (Pro)	55.7; 54.6; 14.1	50.4; 49.6; 10.2	57.2; 59.7; 12.0	51.6; 49.6; 10.3	0.253
valine (Val)	43.3; 44.3; 8.1	45.4; 44.3; 8.2	40.7; 38.8; 7.2	44.1; 44.3; 7.9	0.153
methionine (Met)	5.7; 6.3; 1.7	4.9; 4.8; 1.6	4.2; 3.4; 2.3	4.6; 4.8; 1.4	0.033
tyrosine (Tyr)	16.5; 15.6; 3.6	15.2; 14.2; 3.8	17.9; 17.1; 5.1	15.5; 14.2; 4.5	0.126
leucine (Leu)	25.0; 22.4; 7.6	22.8; 23.4; 3.7	17.8; 17.1; 3.9	21.8; 21.4; 4.2	0.0004
isoleucine (Ile)	19.5; 19.6; 3.6	17.8; 17.1; 3.2	16.6; 17.1; 4.2	17.5; 17.1; 3.6	0.146
phenyloalanine (Phe)	47.3; 49.6; 20.9	42.9; 44.3; 14.2	37.2; 38.8; 7.3	41.6; 38.8; 13.2	0.334
tryptophan (Trp)	28.3; 22.4; 13.9	27.2; 26.1; 8.8	22.9; 21.4; 4.1	27.4; 26.1; 10.0	0.209
glutathione (GSH)	53.6; 0.001; 136.3	81.6; 0.001; 156.5	164.2; 0.001; 192.3	73.9; 0.001; 149.9	0.400
gamma-aminobutyric acid (GABA)	0.5; 0.7; 0.5	0.4; 0.2; 0.3	0.3; 0.2; 0.3	0.8; 0.2; 2.6	0.102
para-aminobenzoic acid (PABA)	5.9; 5.5; 3.2	4.9; 4.8; 3.8	16.4; 6.3; 34.6	11.4; 4.8; 35.7	0.084
kynurenine (Kyn)	7.9; 7.4; 4.0	7.2; 6.3; 2.9	5.5; 4.8; 2.9	6.9; 6.3; 2.9	0.080
inosine (Ino)	0.2; 0.001; 0.5	0.3; 0.2; 0.5	0.2; 0.1; 0.3	0.3; 0.001; 0.6	0.560
citrulline (Cit)	0.4; 0.001; 1.6	0.1; 0; 1.2	2.2; 0.001; 3.6	0.9; 0.001; 2.9	0.021
neopterin (Neo)	7.2; 4.1; 5.1	6.9; 6.3; 3.8	5.0; 3.4; 4.1	7.0; 4.8; 5.2	0.135
pyridoxal	0.001; 0.001; 0.003	0; 0; 0.002	0; 0; 0	0; 0; 0	0.328
pyroglutamic acid (Glp)	110.4; 99.8; 32.2	107.0; 99.8; 29.1	83.9; 83.6; 29.0	104.3; 99.8; 27.5	0.030
isopyridoxal	0.001; 0.001; 0.003	0; 0; 0.002	0; 0; 0	0; 0; 0	0.328
glycyl-L-valine	2.0; 1.9; 0.8	1.9; 1.9; 0.8	1.1; 0.7; 0.9	1.7; 1.9; 0.8	0.003

Supplementary Table S2. Mean (M), median (Me) and standard deviation (SD) of metabolites measured in *M.tb*-stimulated whole blood QFT cultures in each group, together with the p-value (uncorrected) of the Kruskal-Wallis test for equality of location parameter across study groups

Metabolite	TB	LTBI	NMP	HC	p-value
	M; Me; SD	M; Me; SD	M; Me; SD	M; Me; SD	
OFT TB1 culture					
lysine (Lys)	22.4; 21.8; 2.5	22.8; 22.0; 2.0	21.9; 21.7; 1.4	22.5; 21.9; 2.4	0.416
arginine (Arg)	0; 0; 0	0; 0; 0	0; 0; 0	0.07; 0; 0.1	0.896
cystine	0; 0; 0	0; 0; 0	0; 0; 0.02	0.01; 0; 0.1	0.334
asparagine (Asn)	22.3; 19.5; 24.8	23.3; 22.4; 5.5	25.4; 25.1; 5.2	23.3; 22.8; 4.6	0.096
glycine (Gly)	26.3; 19.5; 24.8	22.8; 22.3; 16.7	18.8; 20.1; 10.3	20.7; 16.3; 24.9	0.287
serine (Ser)	3.1; 0; 11.6	1.8; 0; 7.1	0; 0; 0	1.6; 0; 10.5	0.576
alanine (Ala)	86.3; 88.3; 19.7	93.1; 92.1; 23.7	87.5; 89.9; 25.9	93.1; 87.3; 32.5	0.875
glutamine (Gln)	50.0; 47.3; 14.5	50.6; 49,8; 10.4	63.0; 65.0; 21.9	52.5; 48.8; 13.7	0.789
hydroxyproline (Hyp)	7.0; 7.1; 1.6	9.4; 9.3; 2.5	6.8; 6.5; 1.7	8.9; 8.4; 2.5	0.00008
threonine (Thr)	239.5; 250.7; 73.1	258.3; 257.4; 20.6	249.0; 246.5; 13.1	255.4; 249.4; 23.6	0.467
cysteine (Cys)	106.0; 105.9; 0.6	101.7; 106.1; 21.9	93.2; 106.2; 36.4	103.1; 106.1; 18.6	0.732
proline (Pro)	51.9; 51.9; 12.3	48.1; 45.9; 12.2	60.4; 62.5; 20.6	49.1; 45.2; 12.7	0.021
valine (Val)	42.4; 43.0; 5.3	46.2; 45.2; 10.9	41.3; 41.3; 10.2	45.9; 44.8; 9.7	0.359
methionine (Met)	4.7; 4.4; 1.0	4.5; 4.3; 1.7	4.3; 4.0; 1.4	4.3; 4.0; 1.7	0.374
tyrosine (Tyr)	15.2; 14.4; 3.6	15.5; 15.2; 4.7	18.3; 17.7; 6.7	15.9; 14.8; 5.6	0.421
leucine (Leu)	25.5; 20.5; 11.6	25.5; 24.5; 8.7	20.3; 20.1; 7.0	23.7; 21.8; 10.0	0.092
isoleucine (Ile)	19.6; 20.4; 2.8	19.9; 19.1; 4.9	19.1; 18.7; 5.1	19.3; 18.6; 4.8	0.788
phenyloalanine (Phe)	43.8; 45.5; 12.9	44.5; 44.1; 12.3	39.7; 40.8; 11.2	42.7; 41.8; 12.5	0.615
tryptophan (Trp)	24.5; 19.4; 11.7	26.1; 23.9; 10.0	22.2; 21.3; 7.2	25.9; 23.3; 9.7	0.616
glutathione (GSH)	116.2; 0; 295.3	123.7; 0; 295.4	305.0; 0; 406.7	160.1; 0; 324.7	0.295
gamma-aminobutyric acid (GABA)	0.08; 0.08; 0.08	0.1; 0.1; 0.09	0.2; 0.04; 0.5	0.2; 0.1; 1.3	0.055
para-aminobenzoic acid (PABA)	4.5; 3.2; 2.9	3.8; 3.0; 3.1	16.4; 7.8; 31.8	9.2; 4.9; 28.4	0.003
kynurenine (Kyn)	7.6; 8.0; 2.7	9.5; 8.7; 3.6	6.0; 5.1; 3.3	7.1; 6.9; 2.4	0.00003
inosine (Ino)	0.1; 0; 0.2	0.3; 0.06; 0.6	0.6; 0.4; 0.8	0.3; 0; 0.6	0.151
citrulline (Cit)	0.5; 0; 2.1	0; 0; 0	2.4; 0; 3.7	0.8; 0; 2.4	0.127
neopterin (Neo)	5.7; 4.6; 3.8	8.2; 6.9; 6.2	5.6; 2.8; 5.0	7.0; 3.9; 5.5	0.729
pyridoxal	0; 0; 0	0; 0; 0	0; 0; 0	0; 0; 0	0.464
pyroglutamic acid (Glp)	77.5; 80.7; 18.3	89.8; 88.3; 18.6	78.4; 79.8; 22.4	86.8; 81.6; 22.1	0.153
isopyridoxal	0; 0; 0	0; 0; 0	0; 0; 0	0; 0; 0	0.464
glycyl-L-valine	1.4; 1.4; 0.1	1.5; 1.5; 0.1	1.4; 1.4; 0.1	1.5; 1.5; 0.1	0.476
OFT TB2 culture					
lysine (Lys)	22.5; 21.7; 2.8	22.8; 22.0; 2.1	21.7; 21.3; 1.3	22.4;21.9; 2.4	0.279
arginine (Arg)	0; 0; 0	0; 0; 0	0; 0; 0	0; 0; 0	0.464
cystine	0; 0; 0	0; 0; 0	0; 0; 0	0.02; 0; 0.2	0.772
asparagine (Asn)	22.3; 22.5; 3.6	22.7; 21.6; 5.1	23.4; 24.2; 4.4	22.3; 22.0; 3.6	0.106

glycine (Gly)	21.4; 16.4; 25.7	23.9; 22.8; 20.5	16.5; 16.3; 10.2	18.8; 15.4; 18.5	0.120
serine (Ser)	3.7; 0; 13.9	1.9; 0; 7.7	0; 0; 0	1.5; 0; 8.8	0.161
alanine (Ala)	83.3; 84.3; 20.5	89.8; 87.8; 21.9	83.2; 80.2; 26.0	88.5; 85.0; 24.1	0.900
glutamine (Gln)	48.9; 44.7; 15.2	49.9; 49.6; 11.1	63.6; 66.4; 23.9	50.7; 47.3; 13.5	0.087
hydroxyproline (Hyp)	7.1; 7.2; 1.2	9.4; 9.1; 2.5	6.9; 6.3; 2.0	8.8; 8.3; 2.3	0.030
threonine (Thr)	245.1; 244.9; 12.2	252.2; 249.4; 17.5	252.2; 246.4; 20.5	248.4; 248.0; 26.4	0.316
cysteine (Cys)	106.1; 105.9; 0.6	99.3; 106.2; 26.8	93.0; 106.1; 36.3	100.5; 106.1; 24.3	0.152
proline (Pro)	51.7; 51.5; 13.0	48.8; 46.4; 11.3	60.2; 63.1; 21.8	48.8; 45.7; 12.7	0.300
valine (Val)	41.6; 42.4; 6.0	45.5; 43.7; 10.5	39.7; 41.5; 10.0	44.3; 43.7; 9.7	0.003
methionine (Met)	4.7; 4.5; 1.0	4.5; 4.5; 1.6	4.0; 3.8; 1.2	4.3; 4.0; 1.3	0.150
tyrosine (Tyr)	14.7; 13.7; 3.4	15.3; 14.7; 4.7	17.2; 16.5; 6.5	15.5; 14.5; 5.3	0.058
leucine (Leu)	25.4; 20.8; 13.2	24.7; 23.7; 8.6	19.0; 18.6; 6.0	22.6; 21.2; 7.5	0.002
isoleucine (Ile)	18.4; 18.6; 2.6	18.8; 18.3; 4.7	17.2; 18.4; 4.5	17.8; 17.3; 4.3	0.52
phenyloalanine (Phe)	41.9; 40.5; 12.9	44.1; 43.9; 12.5	38.6; 40.8; 11.7	41.4; 40.3; 12.9	0.43
tryptophan (Trp)	24.7; 20.4; 11.1	26.0; 23.1; 10.4	22.4; 22.1; 7.2	25.8; 23.0; 10.4	0.106
glutathione (GSH)	174.3; 0; 346.3	162.6; 0; 329.0	355.8; 0; 416.7	185.7; 0; 342.8	0.706
gamma-aminobutyric acid (GABA)	0.1; 0.1; 0.1	0.1; 0.1; 0.1	0.2; 0.1; 0.4	0.3; 0.2; 1.2	0.242
para-aminobenzoic acid (PABA)	4.5; 3.1; 2.8	4.0; 3.2; 3.2	15.7; 7.2; 30.3	9.2; 4.9; 26.0	0.031
kynurenine (Kyn)	6.9; 6.9; 2.3	9.3; 8.5; 3.4	6.1; 5.1; 3.5	6.7; 6.2; 2.4	0.002
inosine (Ino)	0.2; 0; 0.6	0.4; 0.1; 0.7	0.9; 0.6; 1.0	0.4; 0; 0.8	0.240
citrulline (Cit)	0.5; 0; 1.9	0.1; 0; 0.2	2.3; 0; 3.8	0.8; 0; 2.5	0.061
neopterin (Neo)	6.1; 4.1; 4.6	7.6; 7.0; 5.6	5.4; 3.1; 4.7	6.4; 4.2; 4.9	0.237
pyridoxal	0; 0; 0	0; 0; 0	0; 0; 0	0; 0; 0	0.464
pyroglutamic acid (Glp)	79.8; 78.3; 17.9	88.9; 82.6; 21.1	76.7; 75.9; 21.4	86.0; 83.7; 21.6	0.031
isopyridoxal	0; 0; 0	0; 0; 0	0; 0; 0	0; 0; 0	0.464
glycyl-L-valine	1.4; 1.4; 0.1	1.5; 1.4; 0.1	1.5; 1.4; 0.1	1.5; 1.5; 0.1	0.046

Supplementary Table S3. Ion source parameters.

Parameter	Ionization mode	
	positive	negative
Curtain gas (CUR)	30.00 psi	30.00 psi
Ions source voltage (IS)	5500.00 V	-4500.00 V
Temperature (TEM)	500.00°C	500.00°C
Nebuliser gas (GS1)	45.00 psi	45.00 psi
Drying gas (GS2)	45.00 psi	45.00 psi
Entrance potential (EP)	10.00 V	-10.00 V

Supplementary Table S4. Compound-dependent parameters in the targeted scheduled multiple reaction monitoring (sMRM) LC-MS/MS method

Compound	Q1 [Da]	Q3 [Da]	RT [min]	DP [V]	EP [V]	CE [V]	CXP [V]
PABA 1	138	120	0,6	41	10	19	14
PABA 2	138	94	0,6	41	10	17	10
Pyridoxal 1	168,1	94	0,8	16	10	31	14
Pyridoxal 2	168,1	106	0,8	16	10	29	12
Isopyridoxal 1	168,1	138,1	0,8	56	10	39	16
Isopyridoxal 2	168,1	110,1	0,8	56	10	37	12
Inosine 1	269,1	137	2,2	76	10	13	16
Inosine 2	269,1	119,1	2,2	76	10	55	14
Phenylalanine 1	166,1	120,1	2,8	26	10	18	8
Phenylalanine 2	166,1	103	2,8	26	10	37	12
Kynurenine 1	209,1	192,1	2,8	26	10	11	12
Kynurenine 2	209,1	94,1	2,8	26	10	19	12
Neopterin 1	254,1	206,1	2,8	46	10	23	12
Neopterin 2	254,1	190	2,8	46	10	27	22
Isoleucine/leucine 1	132,1	86	2,9	27	10	15	10
Isoleucine/leucine 2	132,1	69,1	2,9	27	10	23	8
Tryptophan 1	205,1	188	2,9	18	10	14	10
Tryptophan 2	205,1	146,1	2,9	18	10	24	7
Methionine 1	150,1	104,1	3	29	10	14	12
Methionine 2	150,1	133,1	3	29	10	12	7
Proline 1	116	70,1	3,2	34	10	18	10
Proline 2	116	68	3,2	34	10	35	8
Valine 1	118,05	72,1	3,2	26	10	13	9
Valine 2	118,05	57,1	3,2	26	10	37	13
Thyrosine 1	182	165	3,2	23	10	12	9
Thyrosine 2	182	91	3,2	23	10	37	11
Pyroglutamic acid	130	56	3,2	46	10	31	26
Cisteine 1	122	59,1	3,3	50	10	24	14
Cisteine 2	122	76,1	3,3	50	10	16	8
Alanine 1	90,1	44	3,4	27	10	13	11
Alanine 2	90,1	45	3,4	27	10	40	10
Hydroxyproline 1	132	86	3,4	25	10	14	9
Hydroxyproline 2	132	68	3,4	25	10	26	8
Threonine	120	57,1	3,4	24	10	18	11
GABA 1	104	87,1	3,4	46	10	13	10
GABA 2	104	69	3,4	46	10	21	8
Glycylo-L-valine 1	175,1	129	3,4	96	10	13	14
Glycylo-L-valine 1	175,1	72	3,4	96	10	23	10
Glycine 1	76	30,1	3,5	21	10	20	14
Glycine 2	76	31	3,5	21	10	43	12
Glutamine 1	147,1	84,1	3,5	30	10	23	10
Glutamine 2	147,1	101,1	3,5	30	10	14	12
Asparagine 1	133,1	87	3,6	25	10	15	10
Asparagine 2	133,1	74,1	3,6	25	10	22	9
Serine 1	106	60,1	3,6	15	10	13	10
Serine 2	106	42	3,6	15	10	28	12
Citrulline 1	176,1	159,1	3,7	16	10	13	10
Citrulline 2	176,1	70,1	3,7	16	10	29	8
GSH 1	308,1	179,1	3,8	26	10	17	10
GSH 2	308,1	162	3,8	26	10	23	16
Arginine 1	175,1	70,2	4,1	26	10	26	8
Arginine 2	175,1	60,1	4,1	26	10	18	8
Lysine 1	147,2	84,2	4,2	24	10	21	10
Lysine 2	147,2	67	4,2	24	10	35	10
Cystine 1	241,1	152	4,2	66	10	21	8
Cystine 2	241,1	73,9	4,2	66	10	47	16

Abbreviations: Q1 - parent ion; Q3 - fragment ion; RT - retention time ; DP - declustering potential; EP - entrance potential; CE - collision energy; CXP - collision cell exit potential

Supplementary Table S5. Linearity and working ranges of the quantitative LC-MS/MS method.

Compound	Working range [ng/ml]	Linearity (p)
lysine (Lys)	25-50	0,99739
arginine (Arg)	5-50	0,99917
cystine	0,5-25	0,99713
asparagine (Asn)	50-500	0,99906
glycine (Gly)	50-500	0,99848
serine (Ser)	5-50	0,99589
alanine (Ala)	0,1-500	0,998
glutamine (Gln)	0,01-50	0,99965
hydroxyproline (Hyp)	5-50	0,99569
threonine (Thr)	100-5000	0,99714
cysteine (Cys)	5-5000	0,99655
proline (Pro)	0,01-50	0,9993
valine (Val)	0,01-50	0,99888
methionine (Met)	0,05-50	0,99893
tyrosine (Tyr)	1-50	0,99594
leucine (Leu)	0,5-50	0,99972
isoleucine (Ile)	05-50	0,99972
phenylalanine (Phe)	0,1-50	0,99953
tryptophan (Trp)	0,01-50	0,99954
glutathione (Gsh)	1000-5000	0,99653
gamma-aminobutyric acid (GABA)	0,1-50	0,99979
para-aminobenzoic acid (PABA)	1-25	0,9982
kynurenine (Kyn)	0,5-50	0,99724
inosine (Ino)	0,5-50	0,99764
citrulline (Cit)	0,5-50	0,99756
neopterin (Neo)	0,1-500	0,99953
pyridoxal	0,1-10	0,99944
pyroglutamic acid (Glp)	5-50	0,99569
isopyridoxal	0,5-10	0,99939
glycyl-L-valine	0,1-50	0,99564
